# Supplementary figures and images for: Mapping factors influencing initiation of antiretroviral treatment among adolescents living with HIV/AIDS in sub-Saharan Africa: A scoping review protocol
Source: PLoS One. 2024 Feb 22;19(2):e0289515. doi: 10.1371/journal.pone.0289515 (PMC10883572; doi:10.1371/journal.pone.0289515)

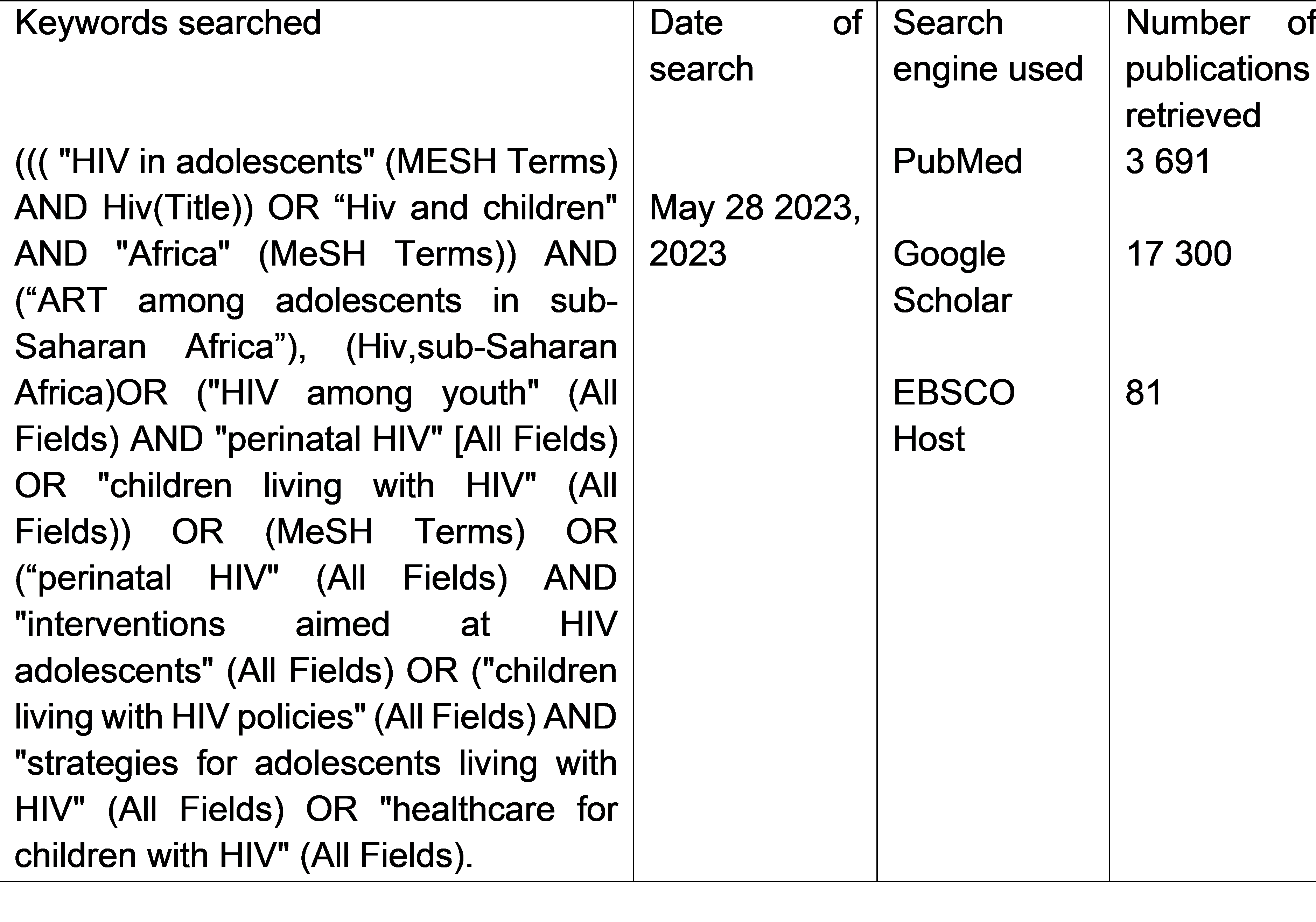

Supplement: S1 Table — (TIF) [file pone.0289515.s002.tif]

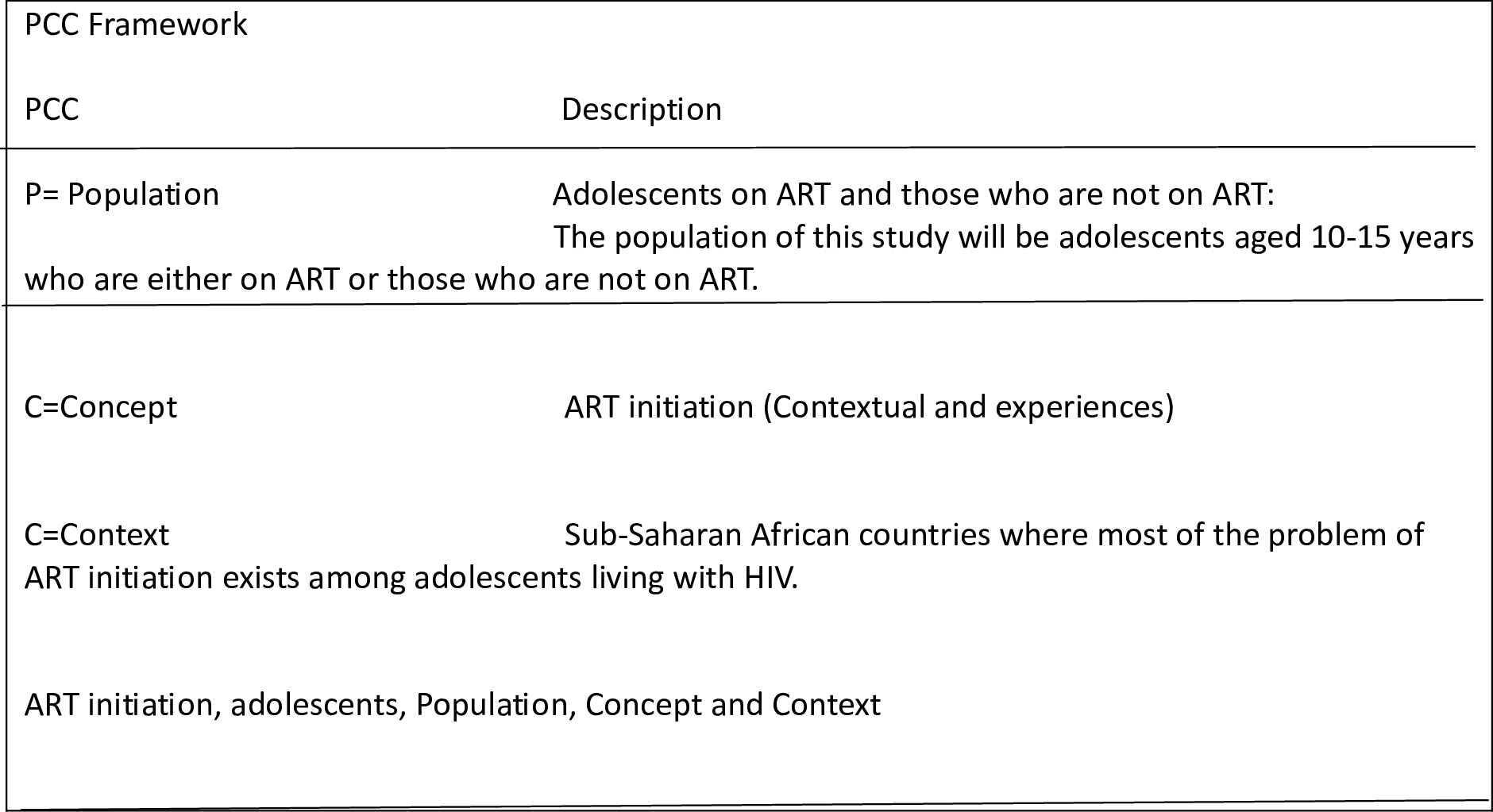

Supplement: S2 Table — (TIF) [file pone.0289515.s003.tif]

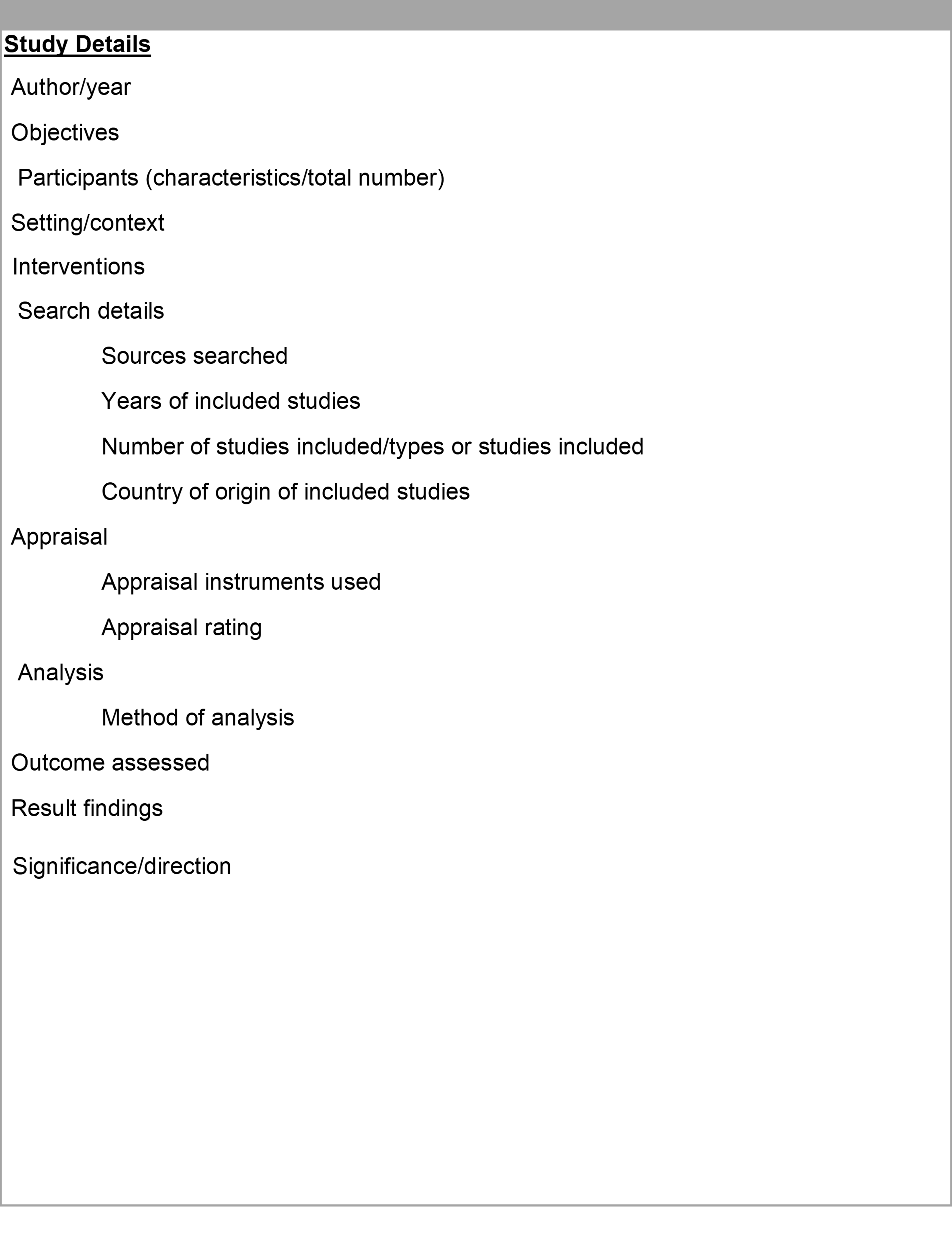

Supplement: S3 Table — (TIF) [file pone.0289515.s004.tif]

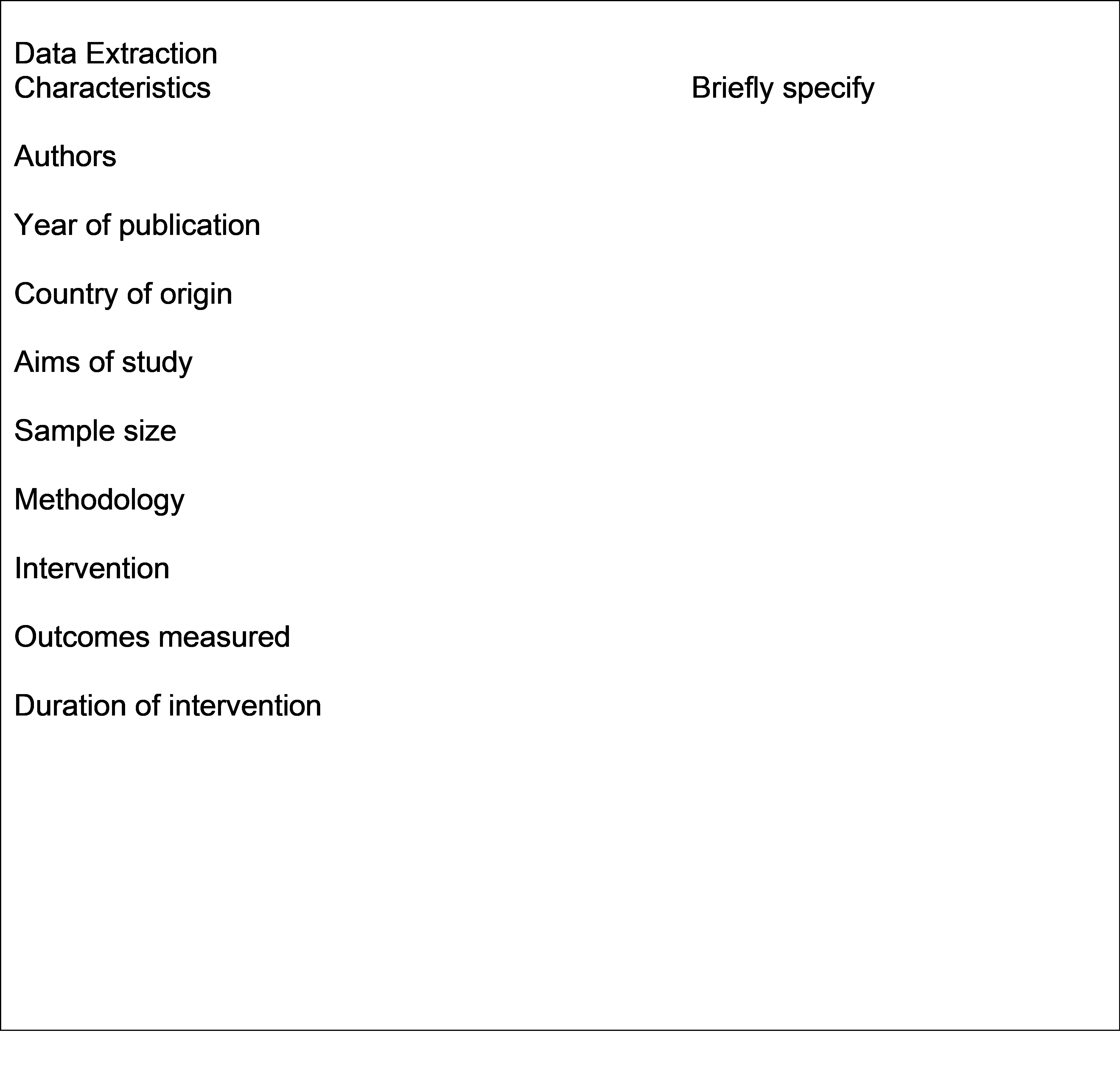

Supplement: S4 Table — (TIF) [file pone.0289515.s005.tif]
