# Supplementary material for: Mapping factors influencing initiation of antiretroviral treatment among adolescents living with HIV/AIDS in sub-Saharan Africa: A scoping review protocol
Source: PLoS One. 2024 Feb 22;19(2):e0289515. doi: 10.1371/journal.pone.0289515 (PMC10883572; doi:10.1371/journal.pone.0289515)
Supplement: Attachment — Submitted filename: PONE-D-23-19003-annotated.pdf [file pone.0289515.s007.pdf]

PLOS ONE

Mapping factors influencing initiation of antiretroviral treatment among adolescents living with HIV/AIDS in sub-Saharan Africa: A scoping review protocol.

--Manuscript Draft--

**Manuscript Number:**	PONE-D-23-19003
**Article Type:**	Study Protocol
**Full Title:**	Mapping factors influencing initiation of antiretroviral treatment among adolescents living with HIV/AIDS in sub-Saharan Africa: A scoping review protocol.
**Short Title:**	Mapping factors influencing initiation of antiretroviral treatment among adolescents living with HIV in Africa
**Corresponding Author:**	Palesa Grace Likoti, PhD<br>University of KwaZulu-Natal College of Health Sciences<br>Durban, KwaZulu-Natal SOUTH AFRICA
**Keywords:**	ART initiation, HIV treatment, adolescents, ART interventions, sub-Saharan Africa, healthcare.
**Abstract:**	Since the Start Free, Stay Free, and AIDS-Free launch, UNAIDS targets intended to promote interventions to prevent HIV transmission and promote access to ART among adolescents and children, of which none were met in 2020. In the sub-Saharan African region(SSA), the number of adolescents initiated on ART drugs remains consistently low. Although HIV prevalence among adolescents has been reduced, several HIV-positive adolescents are dying without being initiated on ART drugs. Therefore, this scoping review protocol aims to map factors influencing the initiation of ART drugs among adolescents living with HIV in SSA.
**Order of Authors:**	Palesa Grace Likoti, PhD
	Nelisiwe Khuzwayo
	Desmond Kuupiel
**Opposed Reviewers:**	
**Additional Information:**	
**Question**	**Response**
**Financial Disclosure**<br><br>Enter a financial disclosure statement that describes the sources of funding for the work included in this submission. Review the submission guidelines for detailed requirements. View published research articles from *PLOS ONE* for specific examples.<br><br>This statement is required for submission and **will appear in the published article** if the submission is accepted. Please make sure it is accurate.	PG Likoti was funded by the University of KwaZulu-Natal, College of Health Sciences PhD scholarship funding. The funders had no role in the analysis or writing of this paper.<br>https://snph.ukzn.ac.za/

Powered by Editorial Manager® and ProduXion Manager® from Aries Systems Corporation

<table>
<tr><td>

Unfunded studies
Enter: *The author(s) received no specific funding for this work.*

Funded studies
Enter a statement with the following details:
• Initials of the authors who received each award
• Grant numbers awarded to each author
• The full name of each funder
• URL of each funder website
• Have or will the sponsors or funders play any role in the study design, data collection (when applicable) and analysis, decision to publish, or preparation of the manuscript?
• **NO** - Include this sentence at the end of your statement: *The funders did not and will not have a role in study design, data collection and analysis, decision to publish, or preparation of the manuscript.*
• **YES** - Specify the role(s) played.

* typeset

</td><td></td></tr>
<tr><td>

Competing Interests

Use the instructions below to enter a competing interest statement for this submission. On behalf of all authors, disclose any competing interests that could be perceived to bias this work—acknowledging all financial support and any other relevant financial or non-financial competing interests.

This statement is required for submission and **will appear in the published article** if the submission is accepted. Please make sure it is accurate and that any funding sources listed in your Funding Information later in the submission form are also declared in your Financial Disclosure statement.

View published research articles from *PLOS ONE* for specific examples.

</td><td>The authors have declared that no competing of interest exits.</td></tr>
</table>

**NO authors have competing interests**<br><br>Enter: *The authors have declared that no competing interests exist.*<br><br>**Authors with competing interests**<br><br>Enter competing interest details beginning with this statement:<br><br>*I have read the journal's policy and the authors of this manuscript have the following competing interests: [insert competing interests here]*<br><br>* typeset	
**Ethics Statement**<br><br>Enter an ethics statement for this submission. This statement is required if the study involved:<br><br>• Human participants<br>• Human specimens or tissue<br>• Vertebrate animals or cephalopods<br>• Vertebrate embryos or tissues<br>• Field research<br><br>Write "N/A" if the submission does not require an ethics statement.<br><br>General guidance is provided below. Consult the submission guidelines for detailed instructions. **Make sure that all information entered here is included in the Methods section of the manuscript.**	This scoping protocol is part of a large-scale study approved by the Biomedical Research Ethics Committee (BREC) of the University of KwaZulu-Natal (BREC Ref No: 0001784/2020). Patients and the public were not involved in the design of this study.

Powered by Editorial Manager® and ProduXion Manager® from Aries Systems Corporation

**Format for specific study types**<br><br>**Human Subject Research (involving human participants and/or tissue)**<br>• Give the name of the institutional review board or ethics committee that approved the study<br>• Include the approval number and/or a statement indicating approval of this research<br>• Indicate the form of consent obtained (written/oral) or the reason that consent was not obtained (e.g. the data were analyzed anonymously)<br><br>**Animal Research (involving vertebrate animals, embryos or tissues)**<br>• Provide the name of the Institutional Animal Care and Use Committee (IACUC) or other relevant ethics board that reviewed the study protocol, and indicate whether they approved this research or granted a formal waiver of ethical approval<br>• Include an approval number if one was obtained<br>• If the study involved *non-human primates*, add *additional details* about animal welfare and steps taken to ameliorate suffering<br>• If anesthesia, euthanasia, or any kind of animal sacrifice is part of the study, include briefly which substances and/or methods were applied<br><br>**Field Research**<br><br>Include the following details if this study involves the collection of plant, animal, or other materials from a natural setting:<br>• Field permit number<br>• Name of the institution or relevant body that granted permission	
**Data Availability**<br><br>Authors are required to make the data underlying their research findings fully available, without restriction.<br><br>If the manuscript reports pilot or preliminary results, the data underlying those results must be made available at the time of publication. A Data Availability	N/A - No results are reported

Statement describing where the data can be found is required at submission. Your answers to this question constitute the Data Availability Statement and will be published in the article, if accepted. When legal or ethical requirements prohibit public data sharing, authors must indicate how researchers may obtain access to the data. See the PLOS Data Policy and FAQ for detailed information.<br><br>**Important:** Stating 'data available on request from the author' is not sufficient. If your data are only available upon request, select 'No' for the first question and explain your situation in the text box.<br><br>For protocols without pilot or preliminary data, authors are strongly encouraged to state how they plan to share research data from their study when it is completed or published.<br><br>If your study includes pilot data, will they be made fully available without restriction?	
**Describe where the data may be found in full sentences. If you are copying our sample text, replace any instances of XXX with the appropriate details.**<br><br>• If **no pilot data are reported** in the manuscript, please enter the following: *No datasets were generated or analysed during the current study. All relevant data from this study will be made available upon study completion.*<br>• If the manuscript reports pilot data **held in a public repository**, include URLs, accession numbers or DOIs. If this information will only be available after acceptance, indicate this by ticking the box below. For example: *All XXX files are available from the XXX database (accession number(s) XXX, XXX.).*<br>• If the manuscript reports pilot data contained **within the manuscript and/or**	Deidentified research data will be made publicly available when the study is completed and published

**Supporting Information files**, enter the following:<br>*All relevant data are within the manuscript and its Supporting Information files.*<br>• If neither of these applies but you are able to provide **details of access elsewhere**, with or without limitations, please do so. For example:<br><br>*Data cannot be shared publicly because of [XXX]. Data are available from the XXX Institutional Data Access / Ethics Committee (contact via XXX) for researchers who meet the criteria for access to confidential data.*<br><br>*The data underlying the results presented in the study are available from (include the name of the third party and contact information or URL).*<br>• This text is appropriate if the data are owned by a third party and authors do not have permission to share the data.<br><br>For study protocols with data management or sharing plans, authors are encouraged to briefly describe in their Data Availability Statement how data they generate will be made accessible when the study is completed. For example:<br>• Deidentified research data will be made publicly available when the study is completed and published.<br><br>* typeset	
Additional data availability information:	

Powered by Editorial Manager® and ProduXion Manager® from Aries Systems Corporation

19 July 2023

1265 Battery Street Suit 200

San Francisco, CA 94111

PlOS ONE

Dear Editor

I wish to submit my original research article for publication on PLOS ONE, titled, “Mapping factors influencing initiation of antiretroviral treatment among adolescents living with HIV/AIDS in sub-Saharan Africa: A scoping review protocol”. The paper was co-authored by Nelisiwe Khuzwayo and Desmond Kuupiel.

A recent study reported that counselling messages during ART initiation should accurately outline treatment risks without perpetuating fear-based narratives among adults living with HIV for them to initiate timely ART. However, evidence on ART initiation among adolescents is limited. In this study, we assessed factors influencing ART initiation among adolescents in sub-Saharan Africa. The study is currently in progress and has not been completed. Upon completion, we believe that our study makes a significant contribution to the literature because there is a need to investigate ART initiation interventions among adolescents living with HIV.

Further, we believe that this paper will be of interest to the readership of your journal because it is a peer reviewed scientific journal. In addition, publishing with PLOS ONE will enable me to share my findings, ideas and new knowledge gained within the academic community. In addition, for publishing fees, I have attached a waiver letter from my university under “other file” and I take responsibility of excess costs. Please consider, as potential editors, Maueen Musimbi Akolo and Jerome T Galea.

This manuscript has not been published and is not under consideration for publication elsewhere, and we have no conflicts of interest to disclose.

Thank you for your consideration.

Sincerely,

Palesa Likoti

University of KwaZulu-Natal, South Africa

Manuscript

Click here to access/download;Manuscript;MAIN MANUSCRIPT.docx

Mapping factors influencing initiation of antiretroviral treatment among adolescents living with HIV/AIDS in sub-Saharan Africa: A scoping review protocol.

Palesa Grace Likoti, Nelisiwe Khuzwayo, Desmond Kuupiel

Corresponding author: Palesa Grace Likoti

parisgracelikoti@gmail.com

School of Nursing and Public Health, University of KwaZulu-Natal

Durban, South Africa

The discipline of Public Health Medicine, School of Nursing and Public Health, University of KwaZulu-Natal, Durban 4041, South Africa

Co-author: Nelisiwe Khuzwayo

Khuzwayone@ukzn.ac.za

School of Nursing and Public Health, University of KwaZulu-Natal

Durban, South Africa

The discipline of Public Health Medicine, School of Nursing and Public Health, University of KwaZulu-Natal, Durban 4041, South Africa.

Co-author: Desmond Kuupiel

Desmondkuupiel98@hotmaail.com

School of Nursing and Public Health, University of KwaZulu-Natal

Durban, South Africa

The discipline of Public Health Medicine, School of Nursing and Public Health, University of KwaZulu-Natal, Durban 4041, South Africa.

Abstract

Background

Since the Start Free, Stay Free, and AIDS-Free launch, UNAIDS targets intended to promote interventions to prevent HIV transmission and promote access to ART among adolescents and children, of which none were achieved in 2020. In the sub-Saharan African region (SSA), the number of adolescents initiated on ART drugs remained consistently low. Although HIV prevalence among adolescents had been reduced, several HIV- positive adolescents died without being initiated on ART drugs. Therefore, this scoping review protocol aims to map factors influencing the initiation of ART drugs among adolescents living with HIV in SSA.

Methods

The methodological framework for scoping reviews will guide this scoping protocol. A search strategy will be used to search literature in electronic databases, including EBSCOhost (PubMed/MEDLINE), Google Scholar, Science Direct, Scopus, BioMed Central, and the World Health Organization library for citations and literature using keywords and the Medical Subjects Heading (MeSH). The electronic databases will be supplemented by hand-searching references on the included studies. The search will be from Jan 01, 2012, to Dec 31, 2022. Articles will be searched and assessed for eligibility by two screeners uploaded on the Endnote software, and duplicates will be identified and removed before the abstract screening. ~~The~~ two screeners will assess the eligibility of the abstracts and the complete articles of the selected studies using the inclusion and exclusion criteria. A third screener will intervene when disputes arise between the two screeners. The selection process will be documented

by following and using the PRISMA flow diagram. A thematic content analysis will present a narrative account of the extracted data.

Discussion

The results of this review will identify and describe factors influencing the initiation of Antiretroviral treatment among adolescents living with HIV in the Sub-Saharan Africa region. The findings will guide future research and inform tailored interventions and strategies for initiating ART among adolescents.

Registration: Open Science Framework. https://doi.org/10.17605/OSF.IO/RNF2T

Keywords: ART initiation, HIV treatment, adolescents, ART interventions, sub-Saharan Africa, healthcare.

Strengths and limitations of this study

- This scoping review will provide insight into factors influencing ART initiation among adolescents living with HIV/AIDS. This is due to limited synthesis and research on factors influencing ART initiation among this population in the sub-Saharan region. Through the review process, we will identify the current literature on this subject to inform future studies when developing ART initiation interventions for adolescents and contribute to a broad understanding of the topic.

- This review is limited to studies written in English-language and published in the last ten years. Non-English studies conducted on ART initiation among adolescents may need to be included. This review is limited to the sub-Saharan region and may not be generalisable to other similar settings, but we anticipate it will provide rich data for future studies. Moreover, we will attempt

to conduct an extensive search of published and grey literature. However, our review may only partially capture the body of evidence on this subject area.

- It is possible that our review will not include all articles published in every journal since some may not be accessible.

Background

Worldwide an estimated 1.8 million adolescents between the ages of 10 and 19 were reported to be living with HIV by the end of 2020 [1]. Approximately 90% of them reside in the sub-Saharan region, which is the epicentre of HIV [2]. Globally, approximately 54% of adolescents aged 10-19 received ART in 2021[1]. There was no significant difference in ART coverage between adolescent girls (58%) and boys (60%), respectively [3].

In 2021 alone, approximately 110 000 children and adolescents died from AIDS-related complications in 2021[4]. Since there is no cure for HIV/AIDS, primary interventions significantly prevent HIV ~~infections.~~ Although these interventions are prevalent in the sub-Saharan region, HIV infection among adolescents aged 10-19 is still high. HIV treatment has been scientifically proven to save lives if accessed early and used appropriately; yet several adolescents succumb to AIDS-related complications annually [5].

In sub-Saharan Africa, numerous interventions promote access to HIV treatment, including primary prevention and HIV treatment literacy; most of these interventions target older people [6]. A study conducted in South Africa revealed sizable gaps in the uptake of critical HIV services targeting young people [7]. These are congruent with a study conducted in Zimbabwe that found a growing number of new infections among adolescents and young people [8]. Hence, significantly shaping the HIV epidemic. Nevertheless, most HIV programs and other health services have been primarily orientated toward adults, with limited commitment to the specific needs of children and adolescents [9].

While programmes to prevent new HIV infections among people with HIV have been hugely successful in recent years, low HIV testing, diagnosis and treatment initiation has been slow and more difficult to achieve among young people (15-24 years) in sub-Saharan Africa [10]. To close the gaps in HIV treatment and prevention, the Joint United Nations Programme on HIV/AIDS (UNAIDS) adopted ambitious, fast-track targets aimed at getting 90% of people living with HIV to know their HIV status, 90% of people to receive treatment and 90% of people on treatment to be virally suppressed. Driven by the concern that these targets were not achieved in 2020, the targets have been revised and increased to 95% by 2025. Many countries in the sub-Saharan region are far from achieving 95-95-95 by 2025 [11].

Treatment strategies of 95-95-95 targets revealed various challenges prohibiting meaningful access to treatment and favourable health outcomes among young people [12]. Some challenges include unprepared health systems that address unique adolescents' needs and factors such as stigma and discrimination resulting in adolescents' reluctance to take ART [13]. Therefore, poor ART coverage among adolescents is limited to access to HIV testing and non-adherence, which decreases viral load suppression among adolescents compared to adults [14].

A framework was launched in 2015 by the Joint United AIDS, aiming to end HIV/AIDS among children, adolescents, and young women, specifically focusing on sub-Saharan Africa [15]. The target set to be reached by 2020 was to Start Free, Stay Free and be AIDS-Free; though, none was met in 2020. For instance, the stay-free target of reducing the number of new HIV infections among adolescents and young women (aged 15-24 years) to less than 100 000 fell short. The World Health Organisation reported approximately 1 100 new HIV infections daily among

adolescents aged 15-24 in 2022 in the sub-Saharan region [16]. While estimates of new HIV infections are staggering, the AIDS-free target aiming at providing 1.4 million children aged 0-14 years and one million adolescents(aged-15-19) HIV treatment was not reached, and more than half (740 00) children were reached by 2020, only 480 000 adolescents were initiated in ART [17].

Since these targets were not reached, countries implemented HIV interventions supporting Start Free, Stay Free, AIDS-Free targets set to be reached by 2030 [18]. Hence, this study aims to map evidence of factors influencing ART initiation among adolescents aged 10-19 years living with HIV in the sub-Saharan region. The study's findings employed healthcare services to revise ART initiation guidelines that will prioritise adolescents' needs and improve healthcare service delivery to accommodate adolescents.

METHODS

This scoping review aims to synthesise evidence on factors influencing ART initiation among adolescents living with HIV/AIDS in sub-Saharan Africa. This scoping review forms part of a PhD project. This scoping review will adopt a framework that was proposed by Arksey and O'Malley, which involves following the steps: Identification of research questions, identification of relevant studies, Selection of the studies, extracting and charting the data, Collating, summarising, and reporting the data [19]. The steps are described in detail below:

Step1. Identifying research question

The main proposed research question for this scoping review is "What evidence on the factors influencing ART initiation exists among adolescents living with HIV in sub-Saharan Africa?"

The sub-questions are:

1. What evidence on the individual factors influencing adolescents' initiation of ARTs in sub-Saharan Africa exists?
2. What evidence exists on family and community factors influencing adolescent initiation of ARTs in sub-Saharan Africa?
3. What evidence of health system factors influencing adolescent ARTs initiation in sub-Saharan Africa exists?

Stage 2: Identification of the relevant studies

We will search for relevant literature focused on adolescents and HIV treatment from PubMed, Web of Science, EBSCOhost (Academic search complete, CINAHL and MEDLINE), Google Scholar, and the World Health Organization library database, as well as other relevant websites that have literature on this study. We will also manually assess reference lists for relevant articles. The team will review and assess the results to ensure the validity of the search strategy. Further, all the results from databases and manual searches will be exported to the Endnote X 9 library, and duplicates will be identified and removed. The search was conducted on Dec 31, 2022 (S 2).

Eligibility criteria

Studies to be included in the review must meet the following criteria:

- Studies with study participants aged 10 – 24 years.
- Studies with evidence of ART treatment among adolescents.

- Studies with evidence on ART initiation interventions among adolescents.
- Studies with evidence about HIV-positive adolescents.
- Studies published from Jan 01, 2012, to Dec 01 2022.
- Peer-reviewed literature, grey literature, government documents, policy briefs, systematic reviews, and meta-analyses.
- Studies conducted in sub-Saharan African countries
- **Exclusion criteria**

The following exclusion criteria will include:

- Studies involving HIV-negative adolescents and young people
- Studies focused on HIV screening and/or testing
- Studies focused on HIV viral suppression.
- Articles published in other languages other than English

3. Selection of eligible studies

The selection of the studies will be summarised using a PRISMA flow chart (Fig 1.). The researchers will pilot the search strategy to assess its appropriateness on the selected electronic databases and the keywords of our study. The principal investigator will conduct the title screening, and two reviewers will do the abstract screening independently. The full-text article screening will be done independently, based on the eligibility criteria. When a disagreement arises between two reviewers, the third reviewer will decide. Where there are no complete copies of articles, the researchers will seek assistance from the University of KwaZulu-Natal (UKZN) library services and contact authors to request complete copies of articles attainable within

the UKZN library database. The articles will be assessed to ensure we can answer the research question. Empirical studies with mixed methods, qualitative and quantitative studies will be included. The other types of literature included are review articles, commentary and dissertations.

Quality assurance of the study

To assess the quality assurance of the study, we will use the Mixed Method Appraisal tool version 2020 (MMAT) [20]. This tool will be employed to examine the quality of an article by looking at the following aspects: the appropriateness of the aim of the study, if it is suitable, the methodology used, the study design, the recruitment of participants, the data collection process, the data analysis, presenting of findings, the discussions by the authors and conclusions. Additionally, the authors will cross-examine the themes obtained and critically examine their relationship in line with the study's research questions. Furthermore, this will be done by examining the meanings of the findings and their relation to the aim of the study and the implications for future studies.

Stage 4: Charting the data

Data from included studies will be captured electronically using a data charting form. The extracted data will include all the variables that can assist the team answer answering the research questions, including the author with date, study design, population, interventions identified, study setting, the significance of findings, and conclusions for the interventions' primary and secondary outcomes.

5: Collating, summarising, and reporting the result

We will use NVIVO software version 10 to code the data from the included studies following the below process:

- Coding data from the selected articles.
- categorising the codes into themes.
- displaying the data.
- identifying critical patterns in the data and identifying sub-themes.
- Summarising and synthesising.

A thematic content analysis will present a narrative account of the extracted data around the following themes: individual factors, family and community factors and health system factors influencing the initiation of antiretrovirals among adolescents. The results will be described about the research question and the study's overall purpose.

DISCUSSION

This scoping review aims to identify and describe factors influencing the initiation of HIV treatment among adolescents. The evidence from the current review may provide a framework that can improve initiating adolescents to ART regime and develop health promotion strategies to enable adolescents living with HIV to seek ART drugs early.

Despite increasing evidence that showed the importance of initiating ART early, high mortality and poor retention prevail among HIV-infected adolescents in sub-Saharan Africa, and young people are still confronted with specific challenges at all stages of the HIV care pathways of diagnosis and ART initiation [21].

The United Joint Nations report on HIV estimates revealed that the highest number of adolescents living with HIV globally are in the sub-Saharan region [22]. Given the existing behavioural interventions and HIV awareness campaigns promoting strategies, young people can use them to protect themselves from HIV infection [23].

It is crucial to consider behavioural and environmental factors in this region that increase the HIV risk for adolescents exposed to sexual assault and some incidences resulting in HIV transmission to monitor the HIV response among this population [24]. While commendable coverage of HIV prevention of mother-to-child transmission among pregnant women living with HIV, some children are born with HIV [25]. Regrettably, understanding the risks and vulnerabilities of children transitioning into adolescents in this region is limited. Less is known about adolescents and how to address their needs for ART initiation and treatment services compared to infants and adults.

Interventions aiming at promoting and enrolling adolescents in ARTs are crucial in saving lives. Most countries in sub-Saharan Africa are implementing a start-free, stay-free, AIDS-free framework to fast-track the eradication of HIV/AIDS among adolescents [18]. Therefore, this study's findings may benefit stakeholders involved in implementing ART interventions and providing other health services targeting adolescents.

List of Abbreviations

ART **-** Antiretroviral Therapy

AIDS**-** acquired immunodeficiency syndrome

HIV- Human Immunodeficiency Virus

UNAIDS- The Joint United Nations Programme on HIV/AIDS

PRISMA- Preferred Reporting Items for Systematic Reviews and Meta-Analyses

SSA-sub**-**Saharan Africa

Declarations

Ethics approval and consent to participate- This scoping protocol is part of a large-scale study approved by the Biomedical Research Ethics Committee (BREC) of the University of KwaZulu-Natal (BREC Ref No: 0001784/2020)**.** Patients and the public were not involved in the design of this study.

Consent for Publication- Not Applicable

Availability of the data materials- Not applicable

Competing Interests-The authors have declared no competing interests.

Funding: This work was funded by the University of KwaZulu-Natal, College of Health Sciences PhD scholarship funding. The funders had no role in the analysis or writing of this paper.

Authors Contribution- PGL conceived and drafted the study as part of her PhD study; NK and DK supervised the protocol development and revisited the manuscript critically for important intellectual content. The three authors are involved in substantial contributions to conception and design. The three authors read and approved the final manuscript. PGL, NK, and DK agreed to be accountable for all aspects of the work.

Acknowledgements- The authors would like to acknowledge the University of KwaZulu-Natal.

Definition of Terms

Adolescent: The World Health Organization has defined adolescence as a unique development phase between childhood and adulthood from 10-19.

Antiretroviral Therapy (ART): This is a collection of three or more drugs given to people infected with HIV to suppress the viral load in their blood. These three drugs suppress HIV to prevent it from replicating.

Adherence: The extent to which a patient's behaviour coincides with the prescribed health care regimen agreed upon through a shared decision-making process between the patient and the health care provider.

Intervention: This is defined as an act or strategy developed for, with, or on behalf of individuals or populations to determine, improve, maintain, promote or modify health systems, functioning, or health conditions.

ART Initiation: This is often defined as a non-emergency intervention and various approaches used to prepare people to begin treatment.

Influencing is the capacity to affect character, behaviour, or development to achieve a particular goal.

Healthcare: Is defined as taking preventative or necessary medical procedures to improve an individual's well-being.

References

1. UNICEF. Global and Regional Trends 2021. (Assessed on May 24, 2023). Available online at: https://data.unicef.org/topic/hivaids/global-regional-trends/.

2. UNAIDS. Global HIV & AIDS statistics—Fact sheet | UNAIDS [Internet]. 2023 [cited 2023 May 27]. Available from: https://www.unaids.org/en/resources/fact-sheet

3. UNICEF. HIV and AIDS in adolescents: Global Summary of HIV Epidemic among Adolescents aged 10–19, 2019: UNICEF 2020 [cited 2023 May 27. Available from: https://data.unicef.org/topic/adolescents/hiv-aids/].

4. UNICEF. HIV prevention and treatment progress for children, adolescents and pregnant women early flat over the past few years. UNICEF 2022 [cited 2023 June 28. Available from:https://www.unicef.org/press-releases/hiv-prevention-and-treatment-progress-children-adolescents-and-pregnant-women-nearly

5. Iyun V, Technau KG, Vinikoor M, Yotebieng M, Vreeman R, Abuogi L, Desmonde S, Edmonds A, Amorissani-Folquet M, Davies MA, IeDEA Collaboration. Variations in the characteristics and outcomes of children living with HIV following universal ART in sub-Saharan Africa (2006–17): a retrospective cohort study. The Lancet HIV. 2021 Apr 28.

6. World Health Organization. Levels and trends in child malnutrition: UNICEF.

7. Fact Sheet 2019 [Internet]. 2019 [cited May 26 2023]. http://www.unaids.org/en/resources/fact-sheet.

8. Eaton, JW, Garnett GP, Takavarasha FR, Mason PR, Robertson L, Schumacher CM, Nyamukapa CA, Gregson S. Increasing adolescent HIV prevalence in Eastern Zimbabwe-evidence of long-term survivors of mother-to-child transmission? PloS one. 2013 Aug 7;8 (8):e70447.

9. Baisley K, Chimbindi N, Mthiyane N, Floyd S, McGrath N, Pillay D, Seeley J, Zuma T, Dreyer J, Gareta D, Smit T. High HIV incidence and low uptake of HIV prevention services: The context of risk for young male adults prior to DREAMS in rural KwaZulu-Natal, South Africa. PLoS One. 2018 Dec 26;13(12):e0208689.

10. Giguère K, Eaton JW, Marsh K, Johnson LF, Johnson CC, Ehui E, Jahn A, Wanyeki I, Mbofana F, Bakiono F, Mahy M. Trends in the knowledge of HIV status and efficiency of HIV testing services in sub-Saharan Africa, 2000–20: a modelling study using survey and HIV testing programme data. The Lancet HIV. 2021 May 1;8(5):e284-93.

11. UNAIDS. Fact sheet 95-95-95 targets. 2021.https://www.unaids.org/sites/default/files/mediaasset/UNADSFactsheet en.pdf. Assessed May 27 2023.

12. Joint United Nations Programme on HIV/AIDS. (2018). Global AIDS update 2018: Miles to go –Closing gaps, breaking barriers, righting injustices. http://www.unaids.org/en/resources/documents/2018/global-aids-update.

13. Mbalinda SN, Bakeera-Kitaka S, Amooti DL, Magongo EN, Musoke P, Kaye DK. Ethical challenges of the healthcare transition to adult antiretroviral therapy (ART) clinics for adolescents and young people with HIV in Uganda. BMC medical ethics. 2021 Dec;22(1):1-4.

14. Kahana SY, Fernandez MI, Wilson PA, Bauermeister JA, Lee S, Wilson CM, Hightow-Weidman LB. Rates and correlates of antiretroviral therapy use and virologic suppression among perinatally and behaviorally infected HIV+ youth linked to care in the United States. Journal of acquired immune deficiency syndromes (1999). 2015 Feb 1;68(2):169.

15. Abuelezam NN, McCormick AW, Surface ED, Fussell T, Freedberg KA, Lipsitch M, Seage GR. Modelling the epidemiologic impact of achieving UNAIDS fast-track 90-90-90 and 95-95-95 targets in South Africa. Epidemiology & Infection. 2019;147.

16. Fast-Track: Ending the AIDS epidemic by 2030 [Internet]. Available from: http://www.unaids.org/sites/default/files/media_asset/JC2686_WAD2014report_en.pdf

17. Khalifa A, Stover J, Mahy M, Idele P, Porth T, Lwamba C. Demographic change and HIV epidemic projections to 2050 for adolescents and young people aged 15-24—global—global health action. 2019 Jan 1;12(1):1662685.

18. UNAIDS. Progress towards the Start Free, Stay Free, AIDS-Free targets. 2020 report. 2020. https://www.unaids.org/sites/default/files/ media_asset/start-free-stay-free-aids-free-2020-progress-report_en.pdf (accessed Sept 15, 2021).

19. Arksey H, O'Malley L. Scoping studies: Towards a methodological framework. International journal of social research methodology. 2005 Feb 1;8(1):19-32.

20. Galvão TF, Tiguman GM, Sarkis-Onofre R. The PRISMA 2020 statement in Portuguese: updated recommendations for reporting systematic reviews. Epidemiologia e Serviços de Saúde. 2023 May 22;31:e2022364.

21. Hong QN, Fàbregues S, Bartlett G, Boardman F, Cargo M, Dagenais P, Gagnon MP, Griffiths F, Nicolau B, O'Cathain A, Rousseau MC. The Mixed Methods Appraisal Tool (MMAT) version 2018 for information professionals and researchers. Education for Information. 2018 Jan 1;34(4):285-91.

22. Braun V, Clarke V. Using thematic analysis in psychology. Qualitative research in psychology. 2006 Jan 1;3(2):77-101.

23. . The Joint United Nations Programme on HIV and AIDS (UNAIDS), "The GapReport, "2014, http://www.unaids.org/en/resources/documents/2014/20140716_UNAIDS_gap_report.

24. Wadham E, Green C, Debattista J, Somerset S, Sav A. New digital media interventions for sexual health promotion among young people: a systematic review. Sexual health. 2019 Mar 1;16(2):101-23.

25. George G, Cawood C, Puren A, Khanyile D, Gerritsen A, Govender K, Beckett S, Glenshaw M, Diallo K, Ayalew K, Gibbs A. Evaluating DREAMS HIV prevention interventions targeting adolescent girls and young women in high HIV prevalence districts in South Africa: protocol for a cross-sectional study. BMC women's health. 2020 Dec;20(1):1-1.

Other
Click here to access/download;Figure;S 5 Fig 1.png

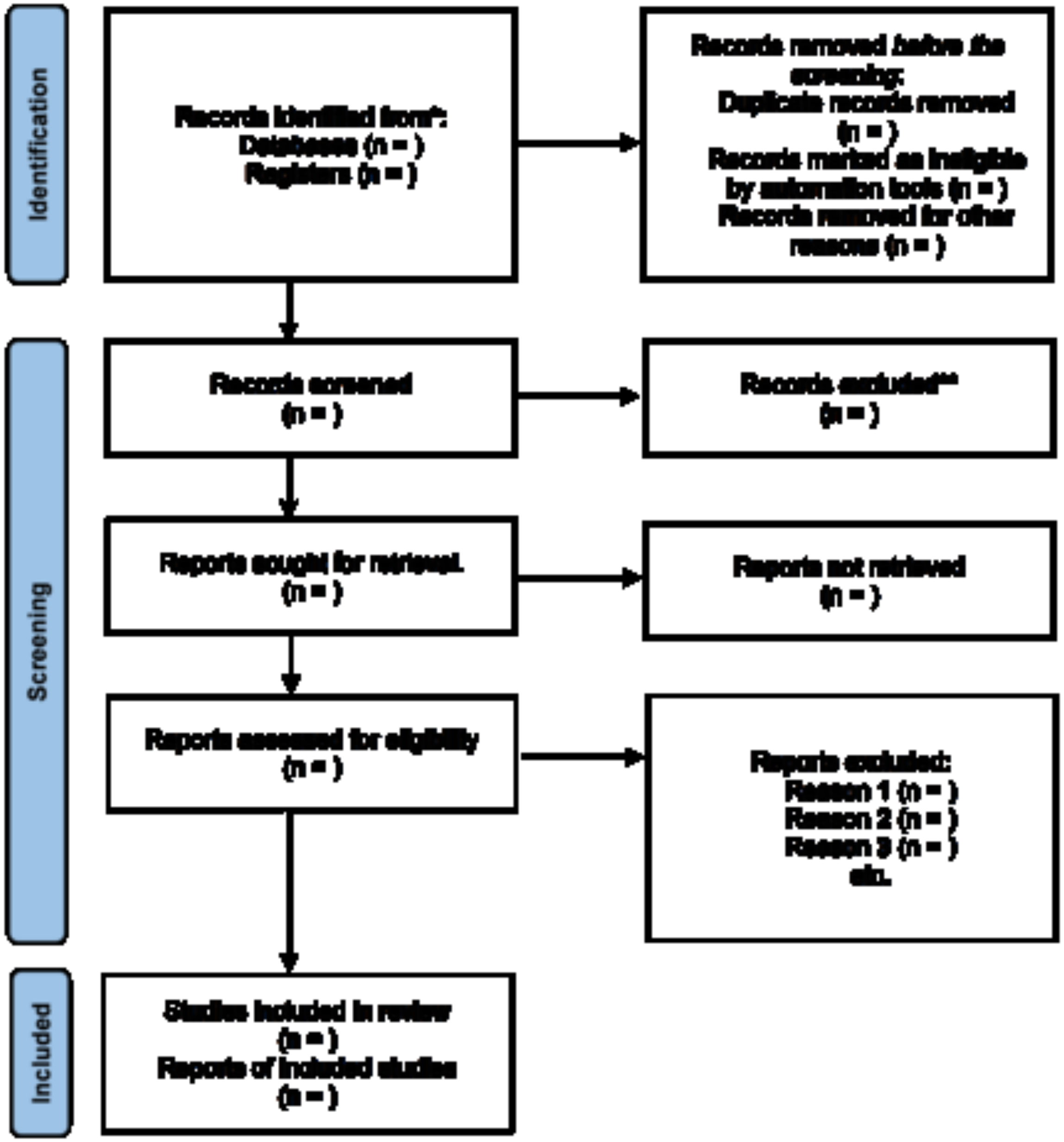

Click here to access/download
Supporting Information
PRISMA-P-checklist.docx

Other

Click here to access/download
Supporting Information
S1.png

Other

Click here to access/download
Supporting Information
S 2.png

Other

Click here to access/download
Supporting Information
S3.png

Other

Click here to access/download
Supporting Information
S4.png

Click here to access/download
Other
PLOS WAIVER REQUEST 2023.pdf

Click here to access/download
Other
MANUSCRIPT WITH TRACK CHANGES.docx

We are grateful for the editor and reviewers for their insightful comments on our paper. We have been able to incorporate changes to reflect most of the suggestions provided by the reviewers and editor. We have responded to the comments and made track changes within the manuscript. Here is a point-by-point response to the editor and reviewers' comments and concerns.

Editor comment: This protocol is a bit confusing as some aspects reflects that the work is completed. Not sure why the investigators would not want to share the review data publicly? Even though it is part of the larger study, but the scoping review is an independent work feeding/linked to the ongoing project. Not sure why the protocol is not registered in PROSPERO? This is important. Could you consider applying the PRISMA-protocol guidelines?

Author response: We thank the editor for these comments. Our study has not yet been completed. All the data supporting the findings will be made available and its supplementary information will be public upon completion of this study. Figures and tables used in this study, along with the original references will be included.

The protocol is registered with Open Science Framework. https://doi.org/10.17605/OSF.IO/RNF2T

The PRISMA checklist has been completed and attached.

1. **Editor comments**: Does the manuscript provide a valid rationale for the proposed study, with clearly identified and justified research questions?

   The research question outlined is expected to address a valid academic problem or topic and contribute to the base of knowledge in the field.

Authors response: We have amended the rational and research questions for the proposed study as seen below:

Rational: This scoping review aims to identify and describe factors influencing the initiation of HIV treatment among adolescents. The evidence from the current review may provide a framework that can improve initiating adolescents to ART regime and

develop health promotion strategies to enable adolescents living with HIV to seek ART drugs early.

Despite increasing evidence that showed the importance of initiating ART early, high mortality and poor retention prevail among HIV-infected adolescents in sub-Saharan Africa, and young people are still confronted with specific challenges at all stages of the HIV care pathways of diagnosis and ART initiation.

Research questions: "

What evidence on the factors influencing ART initiation exists among adolescents living with HIV in sub-Saharan Africa?".

1. What evidence on the individual factors influencing adolescents' initiation of ARTs in sub-Saharan Africa exists?
2. What evidence exists on family and community factors influencing adolescent initiation of ARTs in sub-Saharan Africa?
3. What evidence of health system factors influencing adolescent initiation of ARTs in sub-Saharan Africa exists?

2. **Editor Comments**: The manuscript should describe the methods in sufficient detail to prevent undisclosed flexibility in the experimental procedure or analysis pipeline, including sufficient outcome-neutral conditions (e.g. necessary controls, absence of floor or ceiling effects) to test the proposed hypotheses and a statistical power analysis where applicable. As there may be aspects of the methodology and analysis which can only be refined once the work is undertaken, authors should outline potential assumptions and explicitly describe what aspects of the proposed analyses, if any, are exploratory.

   The manuscript should describe the methods in sufficient detail to prevent undisclosed flexibility in the experimental procedure or analysis pipeline, including sufficient outcome-neutral conditions (e.g. necessary controls, absence of floor or ceiling effects) to test the proposed hypotheses and a statistical power analysis where applicable. As there may be aspects of the methodology and analysis which can only be refined once the work is

undertaken, authors should outline potential assumptions and explicitly describe what aspects of the proposed analyses, if any, are exploratory.

Authors response: We have amended the methodology as follows:

(a)The purpose of our scoping review protocol is to identify knowledge gaps, scope a body of literature of factors that influence ART initiation among adolescents living with HIV in the sub-Saharan region.

(b) The methodological framework for scoping reviews will guide this scoping protocol. A search strategy will be used to search literature in electronic databases, including EBSCOhost (PubMed/MEDLINE), Google Scholar, Science Direct, Scopus, BioMed Central, and the World Health Organization library for citations and literature using keywords and the Medical Subjects Heading (MeSH). The electronic databases will be supplemented by hand-searching references on the included studies. The search will be from Jan 01, 2012, to Dec 31, 2022. Articles will be searched and assessed for eligibility by two screeners uploaded on the Endnote software, and duplicates will be identified and removed before the abstract screening. The two screeners will assess the eligibility of the abstracts and the complete articles of the selected studies using the inclusion and exclusion criteria. A third screener will intervene when disputes arise between the two screeners. The selection process will be documented by following and using the PRISMA flow diagram. A thematic content analysis will present a narrative account of the extracted data.

3. **Editor comment**: Is the methodology feasible and described in sufficient detail to allow the work to be replicable?
   Descriptions of methods and materials in the protocol should be reported in sufficient detail for another researcher to reproduce all experiments and analyses. The protocol should describe the appropriate controls, sample size calculations, and replication needed to ensure that the data are robust and reproducible.

Authors response: We have amended the methodology and how the screening was done as seen below:

METHODS

This scoping review aims to synthesise evidence on factors influencing ART initiation among adolescents living with HIV/AIDS in sub-Saharan Africa. This scoping review forms part of a PhD project. This scoping review will adopt a framework that was proposed by Arksey and O'Malley, which involves following the steps: Identification of research questions, identification of relevant studies, Selection of the studies, extracting and charting the data, Collating, summarising, and reporting the data [19]. The steps are described in detail below:

Step1. Identifying research question

The main proposed research question for this scoping review is “What evidence on the factors influencing ART initiation exists among adolescents living with HIV in sub-Saharan Africa?”.

The sub-questions are:

4. What evidence on the individual factors influencing adolescents’ initiation of ARTs in sub-Saharan Africa exists?
5. What evidence exists on family and community factors influencing adolescent initiation of ARTs in sub-Saharan Africa?
6. What evidence of health system factors influencing adolescent ARTs initiation in sub-Saharan Africa exists?

Stage 2: Identification of the relevant studies

We will search for relevant literature focused on adolescents and HIV treatment from PubMed, Web of Science, EBSCOhost (Academic search complete, CINAHL and MEDLINE), Google Scholar, and the World Health Organization library database, as well as other relevant websites that have literature on this study. We will also manually assess reference lists for relevant articles. The team will review and assess the results to ensure the validity of the search strategy. Further, all the results from databases and manual searches will be exported to the Endnote X 9 library, and duplicates will be identified and removed. The search was conducted on Dec 31, 2022 (S 2).

Eligibility criteria

Studies to be included in the review must meet the following criteria:

- Studies with study participants aged 10 – 24 years.
- Studies with evidence of ART treatment among adolescents.
- Studies with evidence on ART initiation interventions among adolescents.
- Studies with evidence about HIV-positive adolescents.
- Studies published from Jan 01, 2012, to Dec 01 2022.
- Peer-reviewed literature, grey literature, government documents, policy briefs, systematic reviews, and meta-analyses.
- Studies conducted in sub-Saharan African countries
- **Exclusion criteria**

The following exclusion criteria will include:

- Studies involving HIV-negative adolescents and young people
- Studies focused on HIV screening and/or testing
- Studies focused on HIV viral suppression.
- Articles published in other languages other than English

3. Selection of eligible studies

The selection of the studies will be summarised using a PRISMA flow chart (Fig 1.). The researchers will pilot the search strategy to assess its appropriateness on the selected electronic databases and the keywords of our study. The principal investigator will conduct the title screening, and two reviewers will do the abstract screening independently. The full-text article screening will be done independently, based on the eligibility criteria. When a disagreement arises between two reviewers, the third reviewer will decide. Where there are no complete copies of articles, the researchers will seek assistance from the University of KwaZulu-Natal (UKZN) library services and contact authors to request complete copies of articles attainable within the UKZN library database. The articles will be assessed to ensure we can answer the research question. Empirical studies with mixed methods, qualitative and quantitative studies will be included. The other types of literature included are review articles, commentary and dissertations.

Quality assurance of the study

To assess the quality assurance of the study, we will use the Mixed Method Appraisal tool version 2020 (MMAT) [20]. This tool will be employed to examine the quality of an article by looking at the following aspects: the appropriateness of the aim of the study, if it is suitable, the methodology used, the study design, the recruitment of participants, the data collection process, the data analysis, presenting of findings, the discussions by the authors and conclusions. Additionally, the authors will cross-examine the themes obtained and critically examine their relationship in line with the study's research questions. Furthermore, this will be done by examining the meanings of the findings and their relation to the aim of the study and the implications for future studies.

Stage 4: Charting the data

Data from included studies will be captured electronically using a data charting form. The extracted data will include all the variables that can assist the team answer answering the research questions, including the author with date, study design, population, interventions identified, study setting, the significance of findings, and conclusions for the interventions' primary and secondary outcomes.

5: Collating, summarising, and reporting the result

We will use NVIVO software version 10 to code the data from the included studies following the below process:

- Coding data from the selected articles.
- categorising the codes into themes.
- displaying the data.
- identifying critical patterns in the data and identifying sub-themes.
- Summarising and synthesising.

A thematic content analysis will present a narrative account of the extracted data around the following themes: individual factors, family and community factors and health system factors influencing the initiation of antiretrovirals among adolescents.

The results will be described about the research question and the study's overall purpose.

4. **Editor comment**: Have the authors described where all data underlying the findings will be made available when the study is complete?

   The PLOS Data policy requires authors to make all data underlying the findings described in their manuscript fully available without restriction, with rare exception, at the time of publication. The data should be provided as part of the manuscript or its supporting information, or deposited to a public repository. For example, in addition to summary statistics, the data points behind means, medians and variance measures should be available. If there are restrictions on publicly sharing data—e.g. participant privacy or use of data from a third party—those must be specified.

Authors response: All the data supporting the findings will be made available and its supplementary information will be public upon completion of this study. Figures and tables used in this study, along with the original references will be included.

5. **Editor comment**: Is the manuscript presented in an intelligible fashion and written in standard English?
   PLOS ONE does not copyedit accepted manuscripts, so the language in submitted articles must be clear, correct, and unambiguous. Any typographical or grammatical errors should be corrected at revision, so please note any specific errors here.

   Authors response: Thank you for this comment. This manuscript was taken to a language editor and is now presented in an intelligible fashion, and is written in standard English. The research meets all the applicable ethical standards.

REVIEWER 1

1. **Reviewer comment**: The manuscript can be revised to address some grammatical errors. An example of such an error is the inconsistency in the tenses used. In the abstract, within the methods section, there is use of both the past tense and the future tense. Consistency tense usage can improve the flow of the manuscript. Other errors such as word omissions, and typos exist; specific example includes Pg 7, Paragraph 1, line 5- here seems to be a word missing after the word reduction, and still on Pg 7, paragraph 1, line 9- GIV should be HIV and the word 'rates' can be deleted. Please review the whole manuscript to identify such errors as several of them exist.

   Author response: we have taken the manuscript for language editing and have corrected the grammar as seen below.

Abstract : Since the Start Free, Stay Free, and AIDS-Free launch, UNAIDS targets intended to promote interventions to prevent HIV transmission and promote access to ART among adolescents and children, of which none were achieved in 2020. In the sub-Saharan African region (SSA), the number of adolescents initiated on ART drugs remained consistently low. Although HIV prevalence among adolescents had been reduced, several HIV- positive adolescents died without being initiated on ART drugs. Therefore, this scoping review protocol aims to map factors influencing the initiation of ART drugs among adolescents living with HIV in SSA.

Authors response: Page 7 has been amended as follows : Since these targets were not reached, countries implemented HIV interventions supporting Start Free, Stay Free, AIDS-Free targets set to be reached by 2030 [18]. Hence, this study aims to map evidence of factors influencing ART initiation among adolescents aged 10-19 years living with HIV in the sub-Saharan region. The study's findings employed healthcare services to revise ART initiation guidelines that will prioritise adolescents' needs and improve healthcare service delivery to accommodate adolescents.

2. **Reviewer comment**: My second general comment is that the figure needs to be better presented, with a caption. The figure itself is blurry.
   Author response: thank you for this comment. We have replaced this figure with the PRISMA FLOW diagram 2020 version which is clearer as seen below:

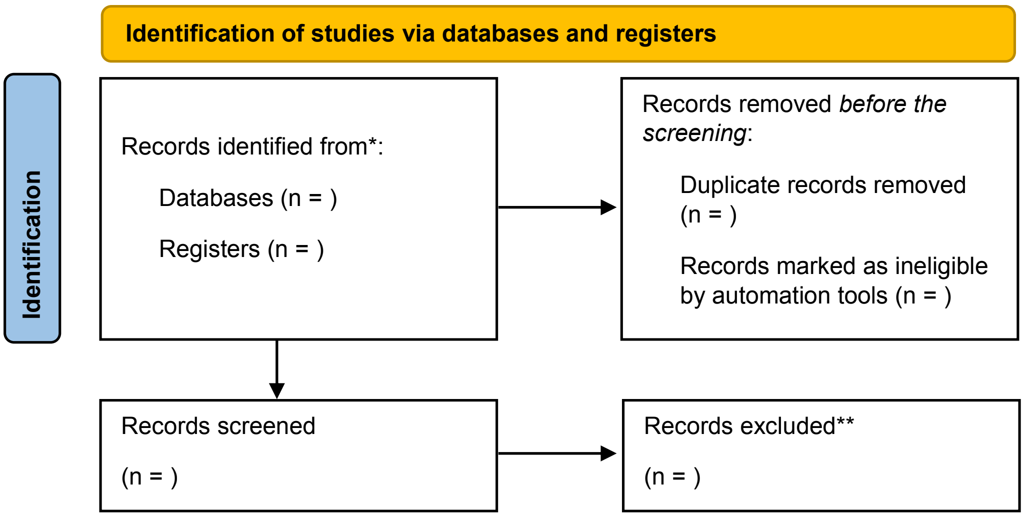

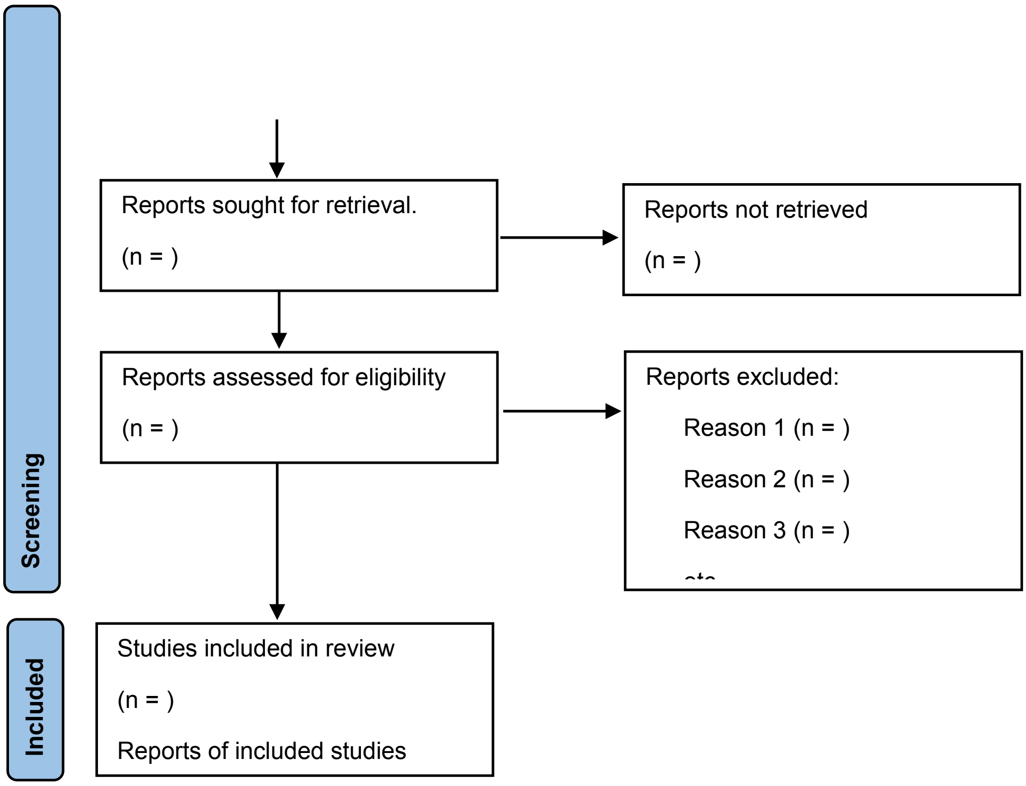

3. **Reviewer comment**: While this is a scoping review protocol which can start off with a very broad question, I would like to suggest you include 'HIV' to the research question in step 1.

Authors response : Thank you for this suggestion, we have amended as follows:

"What are the factors influencing ART initiation among adolescents living with HIV in sub-Saharan Africa?"

4. **Reviewer comment**: Two other thoughts on the methods section:
1. Arskey and O'Malley include the option of a consultation exercise. Could you please provide justification for not including this process in the scoping review

   Authors response: We will consult the key stakeholder groups (health system leaders/decision makers, healthcare providers and service users). We will also consult others who are directly or indirectly involved in the EmONC service delivery so that relevant evidence will be given on literature gaps.

2. What are the differences between Table 3 and Table 4 (stage 4)? Some detail describing the differences and the necessity for both the tables would be helpful for the audience.
3. **Authors response**: The difference between the two tables is that the purpose for table 3 is to create a descriptive summary of the results which address the scoping reviews objectives and answers for the research questions of the review.

   Table 4, The data extraction forms purpose is to link the systematic reviews with primary research to provide the foundation for appraising, analysing, summarising and interpreting a body of evidence. This makes their development, pilot testing and use a crucial part of the systematic review process.
4. **Reviewer comment**: Finally, in the references section, it appears reference 13 needs to be corrected. The first author seems to be missing a last name.

   Author response: Thank you, we have checked our reference and maintain that the first authors name is correct.

REVIEWER 2

1. **Reviewer comment**: The lack of clear, coherent sequential logic in the arguments presented. Often it is difficult for the reader to connect the various references to the literature and data as the arguments are not clearly developed and points not clearly connected (one example being the opening paragraph, which starts with statistics on child mortality and then moves onto stats for adolescent testing, ART initiation, mortality etc without making the link between these. However, there are many further examples)
2.

   Authors response: We have amended this section and updated statistics as seen below:

Worldwide an estimated 1.8 million adolescents between the ages of 10 and 19 were reported to be living with HIV by the end of 2020 [1]. Approximately 90% of them reside in the sub-Saharan region, which is the epicentre of HIV [2]. Globally, approximately 54% of adolescents aged 10-19 received ART in 2021[1]. There was no significant difference in ART coverage between adolescent girls (58%) and boys (60%), respectively [3].

3. **Reviewer comment**: Inappropriate use of language and tenses. For example, a lot of the introduction refers in the past tense to phenomena (e.g. 'there were approx 4 in 5 new infections) without providing a clear time frame or sufficient context for the reader to understand what the authors are referring to. Also, many sentences start with words such as 'similarly', 'in addition', 'moreover' etc where these don't really make sense in the context of the paragraphs or arguments.

Authors response: the entire manuscript has gone through extensive editing and this section has been amended as follows: In 2021 alone, approximately 110 000 children and adolescents died from AIDS-related complications in 2021[4]. Since there is no cure for HIV/AIDS, primary interventions significantly prevent HIV infections. Although these interventions are prevalent in the sub-Saharan region, HIV infection among adolescents aged 10-19 is still high. HIV treatment has been scientifically proven to save lives if accessed early and used appropriately; yet, several adolescents succumb to AIDS-related complications annually [5].

In sub-Saharan Africa, numerous interventions promote access to HIV treatment, including primary prevention and HIV treatment literacy; most of these interventions target older people [6]. A study conducted in South Africa revealed sizable gaps in the uptake of critical HIV services targeting young people [7]. These are congruent with a study conducted in Zimbabwe that found a growing number of new infections among adolescents and young people [8]. Hence, significantly shaping the HIV epidemic. Nevertheless, most HIV programs and other health services have been

primarily orientated toward adults, with limited commitment to the specific needs of children and adolescents [9].

4. **Reviewer comment**: A lot of points included in the discussion should probably have gone into the introduction section (and I'm not sure a discussion even makes sense for a protocol) but, once again, the relevance of a lot of the literature/points cited to the study objectives and gaps to be addressed is unclear

   Authors response: Thank you for this comment. We have made significant amends to the manuscript. The discussion now reads as follows:

This scoping review aims to identify and describe factors influencing the initiation of HIV treatment among adolescents. The evidence from the current review may provide a framework that can improve initiating adolescents to ART regime and develop health promotion strategies to enable adolescents living with HIV to seek ART drugs early.
Despite increasing evidence that showed the importance of initiating ART early, high mortality and poor retention prevail among HIV-infected adolescents in sub-Saharan Africa, and young people are still confronted with specific challenges at all stages of the HIV care pathways of diagnosis and ART initiation [21].

The United Joint Nations report on HIV estimates revealed that the highest number of adolescents living with HIV globally are in the sub-Saharan region [22]. Given the existing behavioural interventions and HIV awareness campaigns promoting strategies, young people can use them to protect themselves from HIV infection [23].

It is crucial to consider behavioural and environmental factors in this region that increase the HIV risk for adolescents exposed to sexual assault and some incidences resulting in HIV transmission to monitor the HIV response among this population [24]. While commendable coverage of HIV prevention of mother-to-child transmission among pregnant women living with HIV, some children are born with HIV [25]. Regrettably, understanding the risks and vulnerabilities of children transitioning into

adolescents in this region is limited. Less is known about adolescents and how to address their needs for ART initiation and treatment services compared to infants and adults.

Interventions aiming at promoting and enrolling adolescents in ARTs are crucial in saving lives. Most countries in sub-Saharan Africa are implementing a start-free, stay-free, AIDS-free framework to fast-track the eradication of HIV/AIDS among adolescents [18]. Therefore, this study's findings may benefit stakeholders involved in implementing ART interventions and providing other health services targeting adolescents.

5. **Reviewer comment**: The focus of this review is unclear. The authors pose as a research question 'what are the contextual factors influencing ART initiation' without clarifying what is meant by contextual factors or providing greater conceptual depth. Other statements in this protocol instead appear to conflict with this aim, e.g. the sentence on pg 12 indicating that the focus is on scope and effectiveness of ART interventions (a very different question).

   Author response: we have amended the research questions as seen below:

The main proposed research question for this scoping review is “What evidence on the factors influencing ART initiation exists among adolescents living with HIV in sub-Saharan Africa ”

The sub-questions are:

1. What evidence on the individual factors influencing adolescents’ initiation of ARTs in sub-Saharan Africa exists?
2. What evidence exists on family and community factors influencing adolescent initiation of ARTs in sub-Saharan Africa?
3. What evidence of health system factors influencing adolescent initiation of ARTs in sub-Saharan Africa exists?

   5.**Reviwer comment**: As a result, the described review methodology is too broad and poorly defined, starting from the search strategy (which is too wide and contains redundant key words in the string) and further highlighted by the

lack of clarity and detail on any sort of conceptual framing, data extraction, how the findings of review papers will be organised and analysed etc.

Author response: Thank you for this comment. We have emended our methodology and search strategies and the overall manuscript as seen below:

METHODS

This scoping review aims to synthesise evidence on factors influencing ART initiation among adolescents living with HIV/AIDS in sub-Saharan Africa. This scoping review forms part of a PhD project. This scoping review will adopt a framework that was proposed by Arksey and O'Malley, which involves following the steps: Identification of research questions, identification of relevant studies, Selection of the studies, extracting and charting the data, Collating, summarising, and reporting the data [19]. The steps are described in detail below:

Step1. **Identifying research question**

The main proposed research question for this scoping review is "What evidence on the factors influencing ART initiation exists among adolescents living with HIV in sub-Saharan Africa "

The sub-questions are:

7. What evidence on the individual factors influencing adolescents' initiation of ARTs in sub-Saharan Africa exists?
8. What evidence exists on family and community factors influencing adolescent initiation of ARTs in sub-Saharan Africa?
9. What evidence of health system factors influencing adolescent ARTs initiation in sub-Saharan Africa exists?

Stage 2: Identification of the relevant studies

We will search for relevant literature focused on adolescents and HIV treatment from PubMed, Web of Science, EBSCOhost (Academic search complete, CINAHL and MEDLINE), Google Scholar, and the World Health Organization library database, as well as other relevant websites that have literature on this study. We will also manually assess reference lists for relevant articles. The team will review and assess the results to ensure the validity of the search strategy. Further, all the results from databases

and manual searches will be exported to the Endnote X 9 library, and duplicates will be identified and removed. The search was conducted on Dec 31, 2022 (S 2).

Eligibility criteria

Studies to be included in the review must meet the following criteria:

- Studies with study participants aged 10 – 24 years.
- Studies with evidence of ART treatment among adolescents.
- Studies with evidence on ART initiation interventions among adolescents.
- Studies with evidence about HIV-positive adolescents.
- Studies published from Jan 01, 2012, to Dec 01 2022.
- Peer-reviewed literature, grey literature, government documents, policy briefs, systematic reviews, and meta-analyses.
- Studies conducted in sub-Saharan African countries
- **Exclusion criteria**

The following exclusion criteria will include:

- Studies involving HIV-negative adolescents and young people
- Studies focused on HIV screening and/or testing
- Studies focused on HIV viral suppression.
- Articles published in other languages other than English

6. Selection of eligible studies

The selection of the studies will be summarised using a PRISMA flow chart (Fig 1.). The researchers will pilot the search strategy to assess its appropriateness on the selected electronic databases and the keywords of our study. The principal investigator will conduct the title screening, and two reviewers will do the abstract screening independently. The full-text article screening will be done independently, based on the eligibility criteria. When a disagreement arises between two reviewers, the third reviewer will decide. Where there are no complete copies of articles, the researchers will seek assistance from the University of KwaZulu-Natal (UKZN) library services and contact authors to request complete copies of articles attainable within the UKZN library database. The articles will be assessed to ensure we can answer

the research question. Empirical studies with mixed methods, qualitative and quantitative studies will be included. The other types of literature included are review articles, commentary and dissertations.

Quality assurance of the study

To assess the quality assurance of the study, we will use the Mixed Method Appraisal tool version 2020 (MMAT) [20]. This tool will be employed to examine the quality of an article by looking at the following aspects: the appropriateness of the aim of the study, if it is suitable, the methodology used, the study design, the recruitment of participants, the data collection process, the data analysis, presenting of findings, the discussions by the authors and conclusions. Additionally, the authors will cross-examine the themes obtained and critically examine their relationship in line with the study's research questions. Furthermore, this will be done by examining the meanings of the findings and their relation to the aim of the study and the implications for future studies.

Stage 4: Charting the data

Data from included studies will be captured electronically using a data charting form. The extracted data will include all the variables that can assist the team answer answering the research questions, including the author with date, study design, population, interventions identified, study setting, the significance of findings, and conclusions for the interventions' primary and secondary outcomes.

5: Collating, summarising, and reporting the result

We will use NVIVO software version 10 to code the data from the included studies following the below process:

- Coding data from the selected articles.
- categorising the codes into themes.
- displaying the data.
- identifying critical patterns in the data and identifying sub-themes.
- Summarising and synthesising.

A thematic content analysis will present a narrative account of the extracted data around the following themes: individual factors, family and community factors and health system factors influencing the initiation of antiretrovirals among adolescents. The results will be described about the research question and the study's overall purpose.
