# Supplementary material for: Mapping factors influencing initiation of antiretroviral treatment among adolescents living with HIV/AIDS in sub-Saharan Africa: A scoping review protocol
Source: PLoS One. 2024 Feb 22;19(2):e0289515. doi: 10.1371/journal.pone.0289515 (PMC10883572; doi:10.1371/journal.pone.0289515)
Supplement: Attachment — Submitted filename: REBUTAL LETTER (AutoRecovered).docx [file pone.0289515.s008.docx]

We are grateful for the editor and reviewers for their insightful comments on our paper. We have responded to the comments from the editor and reviewers. We made track changes within the manuscript. Here is a point-by-point response to the editor and reviewers’ comments and concerns.

1. **Editor comment:** First, the manuscript should be consistent to use sub-Saharan Africa in the text instead of using interchangeably sub-Saharan region and SSA.

**Author response**: We thank the editor for these comments. We have removed “SSA” and remained consistent with sub-Sahara throughout the entire manuscript.

2. **Editor comment**: Second, and this is crucial, the "Limitations of the study" are weakly written. It should answer the following question: "How this paper stands out in the area?". As it stands now, it is unclear of its relevance in the field.

**Author response**: We have amended this section and re wrote it to answer the questions above. The paragraph reads as follows:

**Strengths and limitations of this study**

- This is the first systematic scoping review mapping factors influencing adolescents living with HIV during ART initiation among adolescents in sub-Saharan Africa.
- This scoping review will systematically explore the factors influencing ART initiation among adolescents living with HIV in sub-Saharan Africa and strengthen this evidence through empirical qualitative studies. This is due to limited synthesis and research on factors influencing ART initiation among this population in the sub-Saharan Africa. Through the review process, we will identify the current literature on this subject to inform future studies when developing ART initiation interventions for adolescents and contribute to a broad understanding of the topic.
- Variability in reporting factors influencing ART initiation is expected to be a limitation of this scoping review as there are significant gaps within the literature relating to ART initiation strategies within developing countries.
- This review is limited to studies written in English~~-~~language and published in the last ten years. Non-English studies conducted on ART initiation among adolescents may need to be included. This review is limited to the sub-Saharan Africa and may not be generalisable to other similar settings, but we anticipate it will provide rich data for future studies. Moreover, we will attempt to conduct an extensive search of published and grey literature. However, our review may only partially capture the body of evidence on this subject area.
- It is possible that our review will not include all articles published in every journal since some may not be accessible.

3. **Editor comment:** Third, it is also unclear regarding the age group. Authors refer to young people as 10-24 years or 15-24 years. This needs clarification and consistency in the text.

**Authors response:** Thank you for this comment. We have revised our paper and remained consistent throughout the entire document with the age category 10-19.

4. **Editor comment:** Your ethics statement should only appear in the Methods section of your manuscript. If your ethics statement is written in any section besides the Methods, please move it to the Methods section and delete it from any other section. Please ensure that your ethics statement is included in your manuscript, as the ethics statement entered into the online submission form will not be published alongside your manuscript.

**Author response:** We thank you for this comment. We have moved the ethics statement to methods section a seen below:

**METHODS**

This scoping review aims to synthesise evidence on factors influencing ART initiation among adolescents living with HIV/AIDS in sub-Saharan Africa. This scoping review forms part of a large-scale PhD study approved by the Biomedical Research Ethics Committee (BREC) of the University of KwaZulu-Natal (BREC Ref No: 0001784/2020)**.** Patients and the public were not involved in the design of this study. This scoping review will adopt a framework that was proposed by Arksey and O'Malley, which involves following the steps: Identification of research questions, identification of relevant studies, Selection of the studies, extracting and charting the data, Collating, summarising, and reporting the data [19].

5. **Editor comment :** Please include a caption for figure 1.

**Author response:** Thank you. We have captioned figure 1 as “PRISMA Flow chart (Fig1 File)”

6. **Editor comment:** Please include captions for your Supporting Information files at the end of your manuscript, and update any in-text citations to match accordingly. Please see our Supporting Information guidelines for more information

**Authors response:** Thank you. We have added captions of supporting files in text and at the end of the manuscript as seen below:

**Supporting Information**

**Fig 1** PRISMA FLOW checklist

**S 1** Population-Concept-Context

**S 2** PUB MED search strategy

**S 3** Data Charting Form

**S 4** Mixed Method Appraisal tool version 2020

7. **Reviewer 1 comment :** Some of the statements need to be backed with statistics.

For instance, in the abstract it says 'In the sub-Saharan
African region (SSA), the number of adolescents initiated on ART drugs remained consistently low. Although HIV prevalence among adolescents had been reduced, several HIV- positive adolescents died without being initiated on ART drugs.'

How low is low and what is the data for several ALHIV who have died prior to ART initiation.

**Authors response** :Thank you. We have revised this sentence as follows:

In the sub-Saharan African region, the number of adolescents initiated on ART drugs remained consistently low with approximately100 000 adolescents succumbing to AIDS related causes in 2022.

**8. Reviewer 1 comment**: In abstract methods "A third screener will intervene when disputes arise between the two screeners.' should be reframed to 'A third screener will intervene when there is lack of consensus between the two screeners.'

**Authors response** : Thank you for this comment. We have revied this sentence as seen below: **Methods**

The methodological framework for scoping reviews will guide this scoping protocol. A search strategy will be used to search literature in electronic databases, including EBSCOhost (PubMed/MEDLINE), Google Scholar, Science Direct, Scopus, BioMed Central, and the World Health Organization library for citations and literature using keywords and the Medical Subjects Heading (MeSH). The electronic databases will be supplemented by hand-searching references on the included studies. The search will be from Jan 01, 2012, to Dec 31, 2022. Articles will be searched and assessed for eligibility by two screeners uploaded on the Endnote software, and duplicates will be identified and removed before the abstract screening. The two screeners will assess the eligibility of the abstracts and the complete articles of the selected studies using the inclusion and exclusion criteria. A third screener will intervene when there is lack of consensus between the two screeners. The selection process will be documented by following and using the PRISMA flow diagram. A thematic content analysis will present a narrative account of the extracted data.

**9. Reviewer 1 comment:** In manuscript background 'In 2021 alone, approximately 110 000 children and adolescents died from AIDS-related complications in 2021[4].'

**10. Reviewer 1** comment: Is this a global figure or for SSA?

**Authors response**: Thank you. We have reparsed this sentence as seen below:

Among them, approximately 110 000 children and adolescents died from AIDS-related complications worldwide in 2021[4].

Also 'Although these interventions are prevalent in the sub-Saharan region, HIV infection among adolescents aged 10-19 is still high.'

**11. Reviewer comment 1**: What is the figure?

**Authors response**:	Thank	you	for	this	comment.	We have added the statistics	as	seen	below:

,HIV infection among adolescents aged 10-19 is still high with an estimated 480 000 adolescents newly infected with HIV in 2022 alone [5].

On page 7 'The study' findings recommend healthcare services to revise ART initiation guidelines that will prioritise adolescents' needs and improve healthcare service delivery to accommodate adolescents.'

**12. Reviewer 1 comment:** the sentence is not clear and needs to be revised

**Author response:** 'The study' findings recommend healthcare services to revise ART initiation guidelines that will prioritise adolescents' needs and improve healthcare service delivery to accommodate	adolescents.

**13. Reviewer 1 comment:** On page 9 under inclusion criteria- also include types of studies and articles to be included.

- **Authors response**: Thank you. We have revised this section
  Studies on original research articles reporting on the factors contributing to ART initiation among adolescents living with HIV.
- Studies published in peer-reviewed journals and conducted in sub-Saharan Africa, using any study design that address ART initiation among adolescents living with HIV.

**14. Reviewer 2:** A scoping review of factors influencing initiation of antiretroviral treatment among adolescents living with HIV/AIDS in sub-Saharan Africa: A scoping review protocol

This study aims to address a very important topic that would be a valuable addition to the literature. The planned approach (i.e. search in major databases and using PRISMA flowchart) is appropriate. There are generally accepted approaches to scoping reviews and the authors do follow these. it is not very clear however, where the results will be made available:

**Authors response:** The data supporting this study will be made available upon completion of from the corresponding author upon request.
