# Supplementary material for: Mapping factors influencing initiation of antiretroviral treatment among adolescents living with HIV/AIDS in sub-Saharan Africa: A scoping review protocol
Source: PLoS One. 2024 Feb 22;19(2):e0289515. doi: 10.1371/journal.pone.0289515 (PMC10883572; doi:10.1371/journal.pone.0289515)
Supplement: Attachment — Submitted filename: REVIEWER COMMENTS AND AUTHORS RESPONSE.docx [file pone.0289515.s006.docx]

We are grateful for the editor and reviewers for their insightful comments on our paper. We have been able to incorporate changes to reflect most of the suggestions provided by the reviewers and editor. We have responded to the comments and made track changes within the manuscript. Here is a point-by-point response to the editor and reviewers’ comments and concerns.

**Editor comment**: This protocol is a bit confusing as some aspects reflects that the work is completed. Not sure why the investigators would not want to share the review data publicly? Even though it is part of the larger study, but the scoping review is an independent work feeding/linked to the ongoing project. Not sure why the protocol is not registered in PROSPERO? This is important. Could you consider applying the PRISMA-protocol guidelines?

**Author response**: We thank the editor for these comments. Our study has not yet been completed. All the data supporting the findings will be made available and its supplementary information will be public upon completion of this study. Figures and tables used in this study, along with the original references will be included.

The protocol is registered with Open Science Framework. https://doi.org/10.17605/OSF.IO/RNF2T

The PRISMA checklist has been completed and attached.

1. **Editor comments**: Does the manuscript provide a valid rationale for the proposed study, with clearly identified and justified research questions?

The research question outlined is expected to address a valid academic problem or topic and contribute to the base of knowledge in the field.

**Authors response**: We have amended the rational and research questions for the proposed study as seen below:

**Rational**: This scoping review aims to identify and describe factors influencing the initiation of HIV treatment among adolescents. The evidence from the current review may provide a framework that can improve initiating adolescents to ART regime and develop health promotion strategies to enable adolescents living with HIV to seek ART drugs early.

Despite increasing evidence that showed the importance of initiating ART early, high mortality and poor retention prevail among HIV-infected adolescents in sub-Saharan Africa, and young people are still confronted with specific challenges at all stages of the HIV care pathways of diagnosis and ART initiation.

**Research questions:** “

What evidence on the factors influencing ART initiation exists among adolescents living with HIV in sub-Saharan Africa?”.

1. What evidence on the individual factors influencing adolescents’ initiation of ARTs in sub-Saharan Africa exists?
2. What evidence exists on family and community factors influencing adolescent initiation of ARTs in sub-Saharan Africa?
3. What evidence of health system factors influencing adolescent initiation of ARTs in sub-Saharan Africa exists?
2. **Editor Comments**: The manuscript should describe the methods in sufficient detail to prevent undisclosed flexibility in the experimental procedure or analysis pipeline, including sufficient outcome-neutral conditions (e.g. necessary controls, absence of floor or ceiling effects) to test the proposed hypotheses and a statistical power analysis where applicable. As there may be aspects of the methodology and analysis which can only be refined once the work is undertaken, authors should outline potential assumptions and explicitly describe what aspects of the proposed analyses, if any, are exploratory.

The manuscript should describe the methods in sufficient detail to prevent undisclosed flexibility in the experimental procedure or analysis pipeline, including sufficient outcome-neutral conditions (e.g. necessary controls, absence of floor or ceiling effects) to test the proposed hypotheses and a statistical power analysis where applicable. As there may be aspects of the methodology and analysis which can only be refined once the work is undertaken, authors should outline potential assumptions and explicitly describe what aspects of the proposed analyses, if any, are exploratory.

**Authors response**: We have amended the methodology as follows:

(a)The purpose of our scoping review protocol is to identify knowledge gaps, scope a body of literature of factors that influence ART initiation among adolescents living with HIV in the sub-Saharan region.

(b) The methodological framework for scoping reviews will guide this scoping protocol. A search strategy will be used to search literature in electronic databases, including EBSCOhost (PubMed/MEDLINE), Google Scholar, Science Direct, Scopus, BioMed Central, and the World Health Organization library for citations and literature using keywords and the Medical Subjects Heading (MeSH). The electronic databases will be supplemented by hand-searching references on the included studies. The search will be from Jan 01, 2012, to Dec 31, 2022. Articles will be searched and assessed for eligibility by two screeners uploaded on the Endnote software, and duplicates will be identified and removed before the abstract screening. The two screeners will assess the eligibility of the abstracts and the complete articles of the selected studies using the inclusion and exclusion criteria. A third screener will intervene when disputes arise between the two screeners. The selection process will be documented by following and using the PRISMA flow diagram. A thematic content analysis will present a narrative account of the extracted data.

3. **Editor comment**: Is the methodology feasible and described in sufficient detail to allow the work to be replicable?

Descriptions of methods and materials in the protocol should be reported in sufficient detail for another researcher to reproduce all experiments and analyses. The protocol should describe the appropriate controls, sample size calculations, and replication needed to ensure that the data are robust and reproducible.

**Authors response**: We have amended the methodology and how the screening was done as seen below:

**METHODS**

This scoping review aims to synthesise evidence on factors influencing ART initiation among adolescents living with HIV/AIDS in sub-Saharan Africa. This scoping review forms part of a PhD project. This scoping review will adopt a framework that was proposed by Arksey and O'Malley, which involves following the steps: Identification of research questions, identification of relevant studies, Selection of the studies, extracting and charting the data, Collating, summarising, and reporting the data [19]. The steps are described in detail below:

**Step1**. **Identifying research question**

The main proposed research question for this scoping review is “What evidence on the factors influencing ART initiation exists among adolescents living with HIV in sub-Saharan Africa?”.

The sub-questions are:

4. What evidence on the individual factors influencing adolescents’ initiation of ARTs in sub-Saharan Africa exists?
5. What evidence exists on family and community factors influencing adolescent initiation of ARTs in sub-Saharan Africa?
6. What evidence of health system factors influencing adolescent ARTs initiation in sub-Saharan Africa exists?

**Stage 2: Identification of the relevant studies**

We will search for relevant literature focused on adolescents and HIV treatment from PubMed, Web of Science, EBSCOhost (Academic search complete, CINAHL and MEDLINE), Google Scholar, and the World Health Organization library database, as well as other relevant websites that have literature on this study. We will also manually assess reference lists for relevant articles. The team will review and assess the results to ensure the validity of the search strategy. Further, all the results from databases and manual searches will be exported to the Endnote X 9 library, and duplicates will be identified and removed. The search was conducted on Dec 31, 2022 (S 2).

**Eligibility criteria**

Studies to be included in the review must meet the following criteria:

- Studies with study participants aged 10 – 24 years.
- Studies with evidence of ART treatment among adolescents.
- Studies with evidence on ART initiation interventions among adolescents.
- Studies with evidence about HIV-positive adolescents.
- Studies published from Jan 01, 2012, to Dec 01 2022.
- Peer-reviewed literature, grey literature, government documents, policy briefs, systematic reviews, and meta-analyses.
- Studies conducted in sub-Saharan African countries
- **Exclusion criteria**

The following exclusion criteria will include:

- Studies involving HIV-negative adolescents and young people
- Studies focused on HIV screening and/or testing
- Studies focused on HIV viral suppression.
- Articles published in other languages other than English

3. **Selection of eligible studies**

The selection of the studies will be summarised using a PRISMA flow chart (Fig 1.). The researchers will pilot the search strategy to assess its appropriateness on the selected electronic databases and the keywords of our study. The principal investigator will conduct the title screening, and two reviewers will do the abstract screening independently. The full-text article screening will be done independently, based on the eligibility criteria. When a disagreement arises between two reviewers, the third reviewer will decide. Where there are no complete copies of articles, the researchers will seek assistance from the University of KwaZulu-Natal (UKZN) library services and contact authors to request complete copies of articles attainable within the UKZN library database. The articles will be assessed to ensure we can answer the research question. Empirical studies with mixed methods, qualitative and quantitative studies will be included. The other types of literature included are review articles, commentary and dissertations.

**Quality assurance of the study**

To assess the quality assurance of the study, we will use the Mixed Method Appraisal tool version 2020 (MMAT) [20]. This tool will be employed to examine the quality of an article by looking at the following aspects: the appropriateness of the aim of the study, if it is suitable, the methodology used, the study design, the recruitment of participants, the data collection process, the data analysis, presenting of findings, the discussions by the authors and conclusions. Additionally, the authors will cross-examine the themes obtained and critically examine their relationship in line with the study's research questions. Furthermore, this will be done by examining the meanings of the findings and their relation to the aim of the study and the implications for future studies.

**Stage 4: Charting the data**

Data from included studies will be captured electronically using a data charting form. The extracted data will include all the variables that can assist the team answer answering the research questions, including the author with date, study design, population, interventions identified, study setting, the significance of findings, and conclusions for the interventions' primary and secondary outcomes.

**5: Collating, summarising, and reporting the result**

We will use NVIVO software version 10 to code the data from the included studies following the below process:

- Coding data from the selected articles.

- categorising the codes into themes.

- displaying the data.

- identifying critical patterns in the data and identifying sub-themes.

- Summarising and synthesising.

A thematic content analysis will present a narrative account of the extracted data around the following themes: individual factors, family and community factors and health system factors influencing the initiation of antiretrovirals among adolescents. The results will be described about the research question and the study's overall purpose.

4. **Editor comment**: Have the authors described where all data underlying the findings will be made available when the study is complete?

The [PLOS Data policy](https://journals.plos.org/plosone/s/materials-and-software-sharing) requires authors to make all data underlying the findings described in their manuscript fully available without restriction, with rare exception, at the time of publication. The data should be provided as part of the manuscript or its supporting information, or deposited to a public repository. For example, in addition to summary statistics, the data points behind means, medians and variance measures should be available. If there are restrictions on publicly sharing data—e.g. participant privacy or use of data from a third party—those must be specified.

**Authors response:** All the data supporting the findings will be made available and its supplementary information will be public upon completion of this study. Figures and tables used in this study, along with the original references will be included.

5. **Editor comment**: Is the manuscript presented in an intelligible fashion and written in standard English?

PLOS ONE does not copyedit accepted manuscripts, so the language in submitted articles must be clear, correct, and unambiguous. Any typographical or grammatical errors should be corrected at revision, so please note any specific errors here.

**Authors response**: Thank you for this comment. This manuscript was taken to a language editor and is now presented in an intelligible fashion, and is written in standard English. The research meets all the applicable ethical standards.

**REVIEWER 1**

1. **Reviewer comment**: The manuscript can be revised to address some grammatical errors. An example of such an error is the inconsistency in the tenses used. In the abstract, within the methods section, there is use of both the past tense and the future tense. Consistency tense usage can improve the flow of the manuscript. Other errors such as word omissions, and typos exist; specific example includes Pg 7, Paragraph 1, line 5- here seems to be a word missing after the word reduction, and still on Pg 7, paragraph 1, line 9- GIV should be HIV and the word ‘rates’ can be deleted. Please review the whole manuscript to identify such errors as several of them exist.

**Author response**: we have taken the manuscript for language editing and have corrected the grammar as seen below.

**Abstract :** Since the Start Free, Stay Free, and AIDS-Free launch, UNAIDS targets intended to promote interventions to prevent HIV transmission and promote access to ART among adolescents and children, of which none were achieved in 2020. In the sub-Saharan African region (SSA), the number of adolescents initiated on ART drugs remained consistently low. Although HIV prevalence among adolescents had been reduced, several HIV- positive adolescents died without being initiated on ART drugs. Therefore, this scoping review protocol aims to map factors influencing the initiation of ART drugs among adolescents living with HIV in SSA.

**Authors response**: Page 7 has been amended as follows : Since these targets were not reached, countries implemented HIV interventions supporting Start Free, Stay Free, AIDS-Free targets set to be reached by 2030 [18]. Hence, this study aims to map evidence of factors influencing ART initiation among adolescents aged 10-19 years living with HIV in the sub-Saharan region. The study's findings employed healthcare services to revise ART initiation guidelines that will prioritise adolescents' needs and improve healthcare service delivery to accommodate adolescents.

2. **Reviewer comment**: My second general comment is that the figure needs to be better presented, with a caption. The figure itself is blurry.

**Author response:** thank you for this comment. We have replaced this figure with the PRISMA FLOW diagram 2020 version which is clearer as seen below:

**Identification of studies via databases and registers**

Records removed *before the screening*:

Duplicate records removed (n = )

Records marked as ineligible by automation tools (n = )

Records removed for other reasons (n = )

Records identified from*:

Databases (n = )

Registers (n = )

**Identification**

Records screened

(n = )

Records excluded**

(n = )

Reports sought for retrieval.

(n = )

Reports not retrieved

(n = )

**Screening**

Reports assessed for eligibility

(n = )

Reports excluded:

Reason 1 (n = )

Reason 2 (n = )

Reason 3 (n = )

etc.

Studies included in review

(n = )

Reports of included studies

(n = )

**Included**

3. **Reviewer comment**: While this is a scoping review protocol which can start off with a very broad question, I would like to suggest you include ‘HIV’ to the research question in	step	1.

**Authors response** : Thank you for this suggestion, we have amended as follows:

“What are the factors influencing ART initiation among adolescents living with HIV in sub-Saharan Africa?”

4. **Reviewer comment**: Two other thoughts on the methods section:
1. Arskey and O’Malley include the option of a consultation exercise. Could you please provide justification for not including this process in the scoping review

**Authors response**: We will consult the key stakeholder groups (health system leaders/decision makers, healthcare providers and service users). We will also consult others who are directly or indirectly involved in the EmONC service delivery so that relevant evidence will be given on literature gaps.

2. What are the differences between Table 3 and Table 4 (stage 4)? Some detail describing the differences and the necessity for both the tables would be helpful for the audience.
3. **Authors response**: The difference between the two tables is that the purpose for table 3 is to create a descriptive summary of the results which address the scoping reviews objectives and answers for the research questions of the review.

Table 4, The data extraction forms purpose is to link the systematic reviews with primary research to provide the foundation for appraising, analysing, summarising and interpreting a body of evidence. This makes their development, pilot testing and use a crucial part of the systematic review process.

4. **Reviewer comment**: Finally, in the references section, it appears reference 13 needs to be corrected. The first author seems to be missing a last name.

**Author response**: Thank you, we have checked our reference and maintain that the first authors name is correct.

**REVIEWER 2**

1. **Reviewer comment**: The lack of clear, coherent sequential logic in the arguments presented. Often it is difficult for the reader to connect the various references to the literature and data as the arguments are not clearly developed and points not clearly connected (one example being the opening paragraph, which starts with statistics on child mortality and then moves onto stats for adolescent testing, ART initiation, mortality etc without making the link between these. However, there are many further examples)

**Authors response**: We have amended this section and updated statistics as seen below:

Worldwide an estimated 1.8 million adolescents between the ages of 10 and 19 were reported to be living with HIV by the end of 2020 [1]. Approximately 90% of them reside in the sub-Saharan region, which is the epicentre of HIV [2]. Globally, approximately 54% of adolescents aged 10-19 received ART in 2021[1]. There was no significant difference in ART coverage between adolescent girls (58%) and boys (60%), respectively [3].

3. **Reviewer comment**: Inappropriate use of language and tenses. For example, a lot of the introduction refers in the past tense to phenomena (e.g. 'there were approx 4 in 5 new infections) without providing a clear time frame or sufficient context for the reader to understand what the authors are referring to. Also, many sentences start with words such as 'similarly', 'in addition', 'moreover' etc where these don't really make sense in the context of the paragraphs or arguments.

**Authors response**: the entire manuscript has gone through extensive editing and this section has been amended as follows: In 2021 alone, approximately 110 000 children and adolescents died from AIDS-related complications in 2021[4]. Since there is no cure for HIV/AIDS, primary interventions significantly prevent HIV infections. Although these interventions are prevalent in the sub-Saharan region, HIV infection among adolescents aged 10-19 is still high. HIV treatment has been scientifically proven to save lives if accessed early and used appropriately; yet, several adolescents succumb to AIDS-related complications annually [5].

In sub-Saharan Africa, numerous interventions promote access to HIV treatment, including primary prevention and HIV treatment literacy; most of these interventions target older people [6]. A study conducted in South Africa revealed sizable gaps in the uptake of critical HIV services targeting young people [7]. These are congruent with a study conducted in Zimbabwe that found a growing number of new infections among adolescents and young people [8]. Hence, significantly shaping the HIV epidemic. Nevertheless, most HIV programs and other health services have been primarily orientated toward adults, with limited commitment to the specific needs of children and adolescents [9].

4. **Reviewer comment**: A lot of points included in the discussion should probably have gone into the introduction section (and I'm not sure a discussion even makes sense for a protocol) but, once again, the relevance of a lot of the literature/points cited to the study objectives and gaps to be addressed is unclear

**Authors response**: Thank you for this comment. We have made significant amends to the manuscript. The discussion now reads as follows:

This scoping review aims to identify and describe factors influencing the initiation of HIV treatment among adolescents. The evidence from the current review may provide a framework that can improve initiating adolescents to ART regime and develop health promotion strategies to enable adolescents living with HIV to seek ART drugs early.

Despite increasing evidence that showed the importance of initiating ART early, high mortality and poor retention prevail among HIV-infected adolescents in sub-Saharan Africa, and young people are still confronted with specific challenges at all stages of the HIV care pathways of diagnosis and ART initiation [21].

The United Joint Nations report on HIV estimates revealed that the highest number of adolescents living with HIV globally are in the sub-Saharan region [22]. Given the existing behavioural interventions and HIV awareness campaigns promoting strategies, young people can use them to protect themselves from HIV infection [23].

It is crucial to consider behavioural and environmental factors in this region that increase the HIV risk for adolescents exposed to sexual assault and some incidences resulting in HIV transmission to monitor the HIV response among this population [24].

While commendable coverage of HIV prevention of mother-to-child transmission among pregnant women living with HIV, some children are born with HIV [25]. Regrettably, understanding the risks and vulnerabilities of children transitioning into adolescents in this region is limited. Less is known about adolescents and how to address their needs for ART initiation and treatment services compared to infants and adults.

Interventions aiming at promoting and enrolling adolescents in ARTs are crucial in saving lives. Most countries in sub-Saharan Africa are implementing a start-free, stay-free, AIDS-free framework to fast-track the eradication of HIV/AIDS among adolescents [18]. Therefore, this study's findings may benefit stakeholders involved in implementing ART interventions and providing other health services targeting adolescents.

5. **Reviewer comment**: The focus of this review is unclear. The authors pose as a research question 'what are the contextual factors influencing ART initiation' without clarifying what is meant by contextual factors or providing greater conceptual depth. Other statements in this protocol instead appear to conflict with this aim, e.g. the sentence on pg 12 indicating that the focus is on scope and effectiveness of ART interventions (a very different question).

**Author response**: we have amended the research questions as seen below:

The main proposed research question for this scoping review is “What evidence on the factors influencing ART initiation exists among adolescents living with HIV in sub-Saharan Africa ”

The sub-questions are:

1. What evidence on the individual factors influencing adolescents’ initiation of ARTs in sub-Saharan Africa exists?
2. What evidence exists on family and community factors influencing adolescent initiation of ARTs in sub-Saharan Africa?
3. What evidence of health system factors influencing adolescent initiation of ARTs in sub-Saharan Africa exists?

5.**Reviwer comment**: As a result, the described review methodology is too broad and poorly defined, starting from the search strategy (which is too wide and contains redundant key words in the string) and further highlighted by the lack of clarity and detail on any sort of conceptual framing, data extraction, how the findings of review papers will be organised and analysed etc.

**Author response**: Thank you for this comment. We have emended our methodology and search strategies and the overall manuscript as seen below:

**METHODS**

This scoping review aims to synthesise evidence on factors influencing ART initiation among adolescents living with HIV/AIDS in sub-Saharan Africa. This scoping review forms part of a PhD project. This scoping review will adopt a framework that was proposed by Arksey and O'Malley, which involves following the steps: Identification of research questions, identification of relevant studies, Selection of the studies, extracting and charting the data, Collating, summarising, and reporting the data [19]. The steps are described in detail below:

**Step1**. **Identifying research question**

The main proposed research question for this scoping review is “What evidence on the factors influencing ART initiation exists among adolescents living with HIV in sub-Saharan Africa ”

The sub-questions are:

7. What evidence on the individual factors influencing adolescents’ initiation of ARTs in sub-Saharan Africa exists?
8. What evidence exists on family and community factors influencing adolescent initiation of ARTs in sub-Saharan Africa?
9. What evidence of health system factors influencing adolescent ARTs initiation in sub-Saharan Africa exists?

**Stage 2: Identification of the relevant studies**

We will search for relevant literature focused on adolescents and HIV treatment from PubMed, Web of Science, EBSCOhost (Academic search complete, CINAHL and MEDLINE), Google Scholar, and the World Health Organization library database, as well as other relevant websites that have literature on this study. We will also manually assess reference lists for relevant articles. The team will review and assess the results to ensure the validity of the search strategy. Further, all the results from databases and manual searches will be exported to the Endnote X 9 library, and duplicates will be identified and removed. The search was conducted on Dec 31, 2022 (S 2).

**Eligibility criteria**

Studies to be included in the review must meet the following criteria:

- Studies with study participants aged 10 – 24 years.
- Studies with evidence of ART treatment among adolescents.
- Studies with evidence on ART initiation interventions among adolescents.
- Studies with evidence about HIV-positive adolescents.
- Studies published from Jan 01, 2012, to Dec 01 2022.
- Peer-reviewed literature, grey literature, government documents, policy briefs, systematic reviews, and meta-analyses.
- Studies conducted in sub-Saharan African countries
- **Exclusion criteria**

The following exclusion criteria will include:

- Studies involving HIV-negative adolescents and young people
- Studies focused on HIV screening and/or testing
- Studies focused on HIV viral suppression.
- Articles published in other languages other than English

6. **Selection of eligible studies**

The selection of the studies will be summarised using a PRISMA flow chart (Fig 1.). The researchers will pilot the search strategy to assess its appropriateness on the selected electronic databases and the keywords of our study. The principal investigator will conduct the title screening, and two reviewers will do the abstract screening independently. The full-text article screening will be done independently, based on the eligibility criteria. When a disagreement arises between two reviewers, the third reviewer will decide. Where there are no complete copies of articles, the researchers will seek assistance from the University of KwaZulu-Natal (UKZN) library services and contact authors to request complete copies of articles attainable within the UKZN library database. The articles will be assessed to ensure we can answer the research question. Empirical studies with mixed methods, qualitative and quantitative studies will be included. The other types of literature included are review articles, commentary and dissertations.

**Quality assurance of the study**

To assess the quality assurance of the study, we will use the Mixed Method Appraisal tool version 2020 (MMAT) [20]. This tool will be employed to examine the quality of an article by looking at the following aspects: the appropriateness of the aim of the study, if it is suitable, the methodology used, the study design, the recruitment of participants, the data collection process, the data analysis, presenting of findings, the discussions by the authors and conclusions. Additionally, the authors will cross-examine the themes obtained and critically examine their relationship in line with the study's research questions. Furthermore, this will be done by examining the meanings of the findings and their relation to the aim of the study and the implications for future studies.

**Stage 4: Charting the data**

Data from included studies will be captured electronically using a data charting form. The extracted data will include all the variables that can assist the team answer answering the research questions, including the author with date, study design, population, interventions identified, study setting, the significance of findings, and conclusions for the interventions' primary and secondary outcomes.

**5: Collating, summarising, and reporting the result**

We will use NVIVO software version 10 to code the data from the included studies following the below process:

- Coding data from the selected articles.

- categorising the codes into themes.

- displaying the data.

- identifying critical patterns in the data and identifying sub-themes.

- Summarising and synthesising.

A thematic content analysis will present a narrative account of the extracted data around the following themes: individual factors, family and community factors and health system factors influencing the initiation of antiretrovirals among adolescents. The results will be described about the research question and the study's overall purpose.
